# RNA N6-methyladenosine reader IGF2BP3 interacts with MYCN and facilitates neuroblastoma cell proliferation

**DOI:** 10.1038/s41420-023-01449-3

**Published:** 2023-05-08

**Authors:** Kai Zhu, Tingting Gao, Zhiru Wang, Liaoran Zhang, Kezhe Tan, Zhibao Lv

**Affiliations:** Department of General Surgery, Shanghai Children’s Hospital, School of Medicine, Shanghai Jiao Tong University, Shanghai, PR China

**Keywords:** Oncogenes, Paediatric cancer

## Abstract

Neuroblastoma (NB) is a kind of typical life-threatening extracranial tumor in children. N6-methyladenosine (m6A) modification is closely related to multiple cancer pathological processes. Insulin-like growth factor 2 mRNA binding protein 3 (IGF2BP3) is a top-ranked prognostic risk gene in NB; however, its function is uncertain. The expression of m6A-associated enzymes in patients with NB was analyzed using the Gene Expression Omnibus (GEO) and Therapeutically Applicable Research to Generate Effective Treatments (TARGET) database. The IGF2BP3 level in NB cell lines and primary samples was tested using quantitative real-time polymerase chain reaction (qRT-PCR), western blot method, and immunohistochemical analysis. The IGF2BP3 function in cell proliferation was clarified based on many functional in vitro and in vivo experiments. The interaction between IGF2BP3 and N-myc was researched via RNA immunoprecipitation (RIP), m6A RNA immunoprecipitation (MeRIP), and chromatin immunoprecipitation (ChIP) assays. The 16 m6A-regulated enzymes in NB were researched, and the result indicated that IGF2BP3 overexpression was related to cancer progression, COG risk, and survival based on the GEO and TARGET databases. Besides, the IGF2BP3 and MYCN levels were positively correlated. IGF2BP3 expression levels increased in MYCN-amplified NB clinical samples and cells. Knockdown of IGF2BP3 inhibited N-myc expression and NB cell proliferation in vitro and in vivo. IGF2BP3 regulates MYCN RNA stability by modifying m6A. In addition, we demonstrated that N-myc is a transcription factor that directly promotes IGF2BP3 expression in NB cells. IGF2BP3 regulates the proliferation of NB cells via m6A modification of MYCN. N-myc also acts as a transcription factor that regulates IGF2BP3 expression. A positive feedback loop between IGF2BP3 and N-myc facilitates NB cell proliferation.

## Introduction

Neuroblastoma (NB) is an extracranial solid tumor originating from the neural crest tissue that accounts for approximately 15% of tumor-related mortality in children [1]. NB showed obvious heterogeneity. High-risk tumor patients have a survival rate of less than 50%, even with multimodal therapy, and some patients have spontaneous tumor regression with moderate therapy or without therapy [2]. The pathogenesis of NB is multifactorial and remains unclear. Although traditional clinical risk factors (including tumor stage, age, and histology) have been applied in the field of prognostic risk management and treatment guidance, reliable prognostic molecular indicators are still lacking [3]. So further research on the molecular mechanisms and discovering new prognostic molecular biomarkers are very necessary to improve the treatment of high-risk NB patients.

MYCN contains two closely related genes, C-myc and L-myc, in addition to MYCN (N-myc). Myc family proteins are master regulators of cell fate and are associated with cell growth, senescence, metabolism, and apoptosis [4, 5]. As an important oncogene family member, mutations generally cause carcinogenesis [6]. MYCN gene amplification is a typical genetic marker in NB, which has high application value in predicting the prognosis of such patients [7, 8]. The statistical results show that the prevalence of MYCN amplification in NB patients is 20–30%, and the 5-year overall survival rates were 34.0% for stage 4 patients with MYCN amplification [9, 10]. However, the molecular mechanism of MYCN amplification in the development of NB remains unclear.

In general, the phenotypic characteristics of tumors are regulated by both genetic and epigenetic factors. N6-methyladenosine (m6A) is one of the most intensively studied types in oncology [11, 12]. Several multicenter clinical research have found that single nucleotide mutations (SNPs) in m6A-related enzymes METTL3 [13], METTL14 [14], WTAP [15], YTHDF1 [16], YTHDC1 [17], and YTHDF2 [18] are associated with NB appearance and progression. Another study revealed that miR-98/MYCN axis-mediated NB suppression requires m6A-dependent modification [19]. To determine the correlation between m6A and the pathological process of NB, we analyzed data from the GEO and TARGET databases and found that increased levels of IGF2BP3 were associated with NB prognosis and high-risk phenotypes and that IGF2BP3 and MYCN expression levels were positively correlated. Numerous studies have shown that increased expression of IGF2BP3 is associated with oral squamous cell carcinoma [20], lung cancer [21], melanoma [22], colon cancer [23], liver cancer [24], and gastric cancer [25]. In this study, we showed that IGF2BP3 binds MYCN mRNA with m6A modification and regulates MYCN mRNA stability and expression, and N-myc, in turn, can bind to the IGF2BP3 promoter to promote its expression. IGF2BP3 and MYCN form a positive feedback loop that promotes NB cell proliferation.

## Results

### The expression level of IGF2BP3 increased in high-risk NB cases and was positively associated with MYCN level

**Fig. 1:**
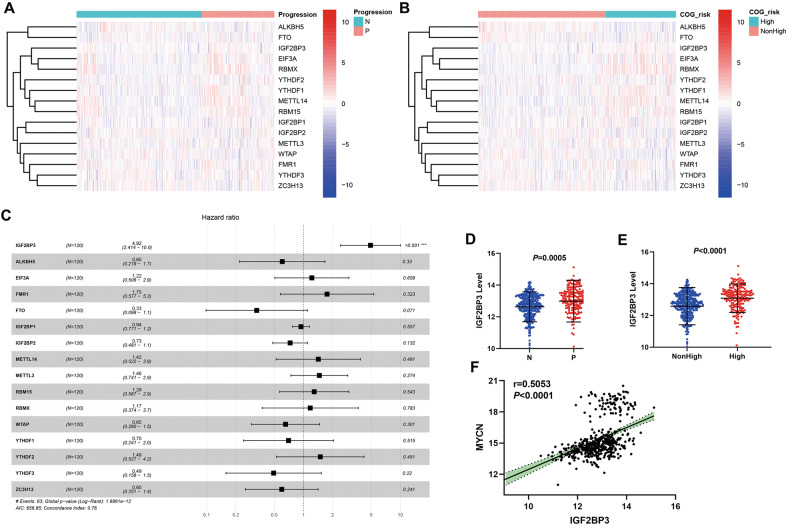
The expression of IGF2BP3 was related to progression and COG risk in NB patients. **A** Differential expression of m6A methylation regulators between subgroups stratified by progression (N: non-progression; P: progression) in dataset GSE49711. **B** Differential expression of m6A methylation regulators between subgroups stratified by COG risk (Children’s Oncology Group risk stratification) in dataset GSE49711. **C** Univariate Cox regression analysis evaluating independently predictive ability of m6A methylation regulators for overall survival of NB patients in TARGET databases. **D** IGF2BP3 expression level in progression and non-progression NB patients of dataset GSE49711. **E** IGF2BP3 expression level in high COG risk and nonhigh COG risk NB patients of dataset GSE49711. **F** The expression levels of IGF2BP3 and MYCN were positively correlated.

To demonstrate the function of m6A methylation regulators in NB development, this paper analyzed their differential expression in important clinicopathological features, tumor progression, and COG risk groups. The results reflected that there exists a close association between m6A regulators and NB clinicopathological features (Fig. 1A, B). To determine prognostic m6A methylation regulators, Cox analysis was conducted for every regulator in the TARGET dataset. IGF2BP3 expression was significantly related to overall survival (Fig. 1C). The expression level of IGF2BP3 in patients with progression of NB was significantly higher than that in patients with non-progression (Fig. 1D). And also, the expression level of IGF2BP3 in patients with high COG risk was significantly higher than that in patients with nonhigh COG risk (Fig. 1E). Meanwhile, according to the analysis result, the IGF2BP3 and MYCN level were positively moderate correlated (Fig. 1F).

### IGF2BP3 expression levels were increased in MYCN-amplified NB clinical samples and cells

**Fig. 2:**
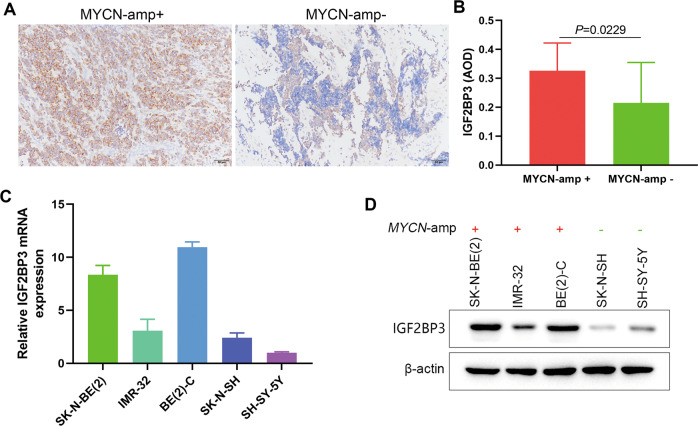
IGF2BP3 is upregulated in MYCN-amplified NB tissues and cell lines. **A** Immunohistochemistry images of IGF2BP3 in MYCN-amplified and non-MYCN-amplified NB tissues (*n* = 35). **B** The expression level of IGF2BP3 in MYCN-amplified tissues was higher than that in non-MYCN-amplified tissues (*n* = 35). **C** qRT-PCR analysis of the expression level of IGF2BP3 in NB cell lines. **D** Western blot analysis of the expression level of IGF2BP3 in NB cell lines (*n* = 3).

Since the function of IGF2BP3 in NB is still unclear, we examined the expression of IGF2BP3 in NB clinical samples and cell lines. Immunohistochemical analysis of 35 NB clinical samples showed that the IGF2BP3 level in MYCN-amplified samples was higher than that in non-MYCN-amplified samples (Fig. 2A, B). Similarly, in NB cell lines, the expression of IGF2BP3 was higher in MYCN-amplified cells. We selected SK-N-BE(2) and BE(2)-C cells with higher IGF2BP3 expression for subsequent observations (Fig. 2C, D).

### Knockdown of IGF2BP3 inhibited N-myc expression and repressed the proliferation of NB cells

**Fig. 3:**
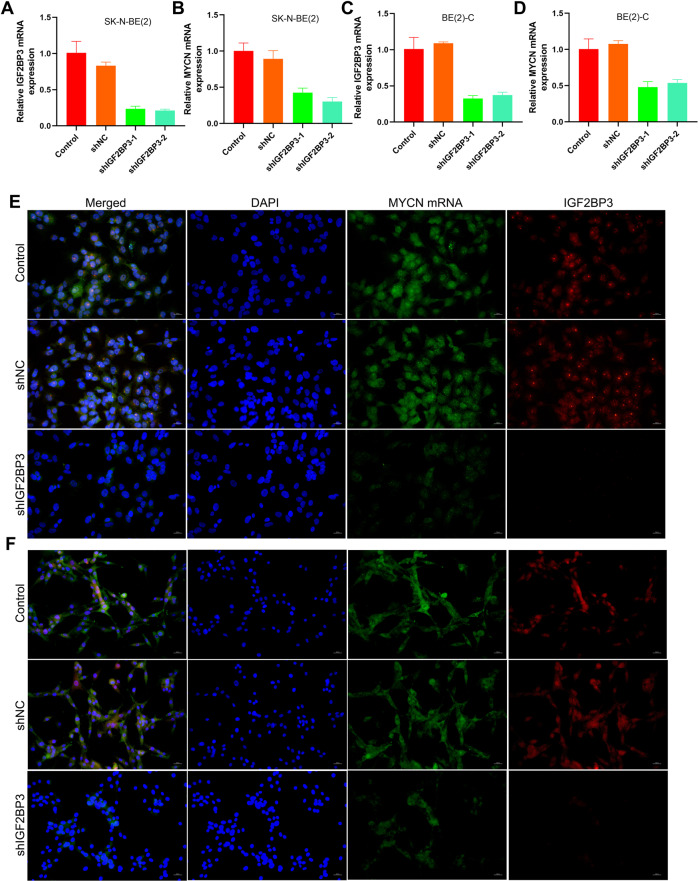
Knockdown of IGF2BP3 inhibited N-myc expression in MYCN-amplified NB cells. **A** qRT-PCR analysis of the expression level of IGF2BP3 mRNA in SK-N-BE(2) (*n* = 3); **B** qRT-PCR analysis of the expression level of MYCN mRNA in SK-N-BE(2) (*n* = 3); **C** qRT-PCR analysis of the expression level of IGF2BP3 mRNA in BE(2)-C (*n* = 3); **D** qRT-PCR analysis of the expression level of MYCN mRNA in BE(2)-C (*n* = 3); **E** RNA-FISH and immunofluorescence analysis of the expression level of MYCN mRNA and IGF2BP3 in SK-N-BE(2) (Green: MYCN mRNA; Red: IGF2BP3 protein; Blue: DAPI); **F** RNA-FISH and immunofluorescence analysis of the expression level of MYCN mRNA and IGF2BP3 in BE(2)-C (Green: MYCN mRNA; Red: IGF2BP3 protein; Blue: DAPI).

**Fig. 4:**
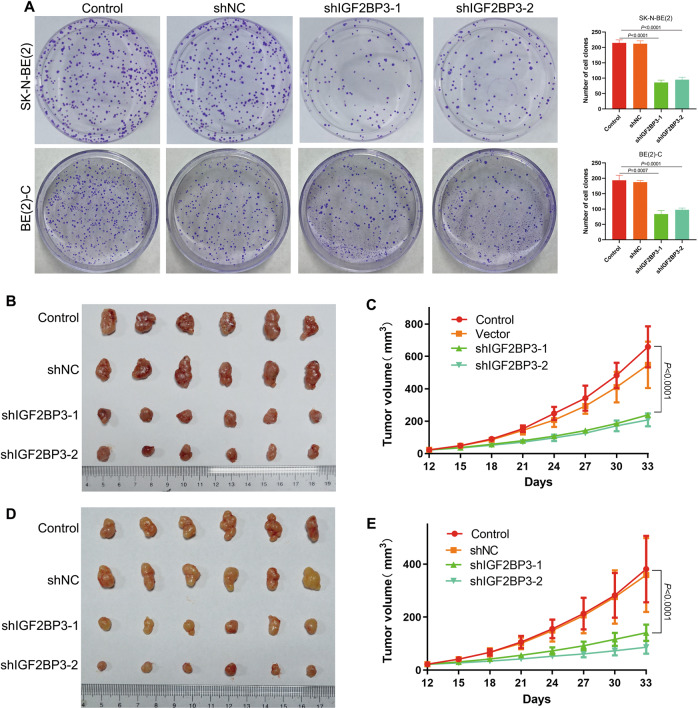
Knockdown of IGF2BP3 repressed proliferation of MYCN-amplified NB cells. **A** Monolayer colonies were detected in SK-N-BE(2) and BE(2)-C (*n* = 3); **B**, **C** Xenograft formation of SK-N-BE(2) cells (*n* = 6); **D**, **E** Xenograft formation of BE(2)-C cells (*n* = 6).

In view of the analysis of the database, IGF2BP3 and MYCN RNA expression levels in NB are positively correlated (Fig. 1F). Next, we downregulate the expression of IGF2BP3 in NB cells and observe the changes in N-myc expression level and biological characteristics. IGF2BP3 was knocked down in SK-N-BE(2)/BE(2)-C cells and confirmed by qRT-PCR (Fig. 3A, C) and western blot methods (Supplementary Fig. S1). Knockdown of IGF2BP3 obviously decreased the level of N-myc, as confirmed by qRT-PCR (Fig. 3B, D), RNA-FISH (Fig. 3E, F), and western blot (Supplementary Fig. S1). Clone formation test showed that knockdown of IGF2BP3 prevented the clonability of SK-N-BE(2) and BE(2)-C cells (Fig. 4A). As expected, IGF2BP3 knockdown inhibited these cells proliferation in nude mice (Fig. 4B–E).

### Repression of m6A activation inhibits N-myc expression and impairs NB cell proliferation

**Fig. 5:**
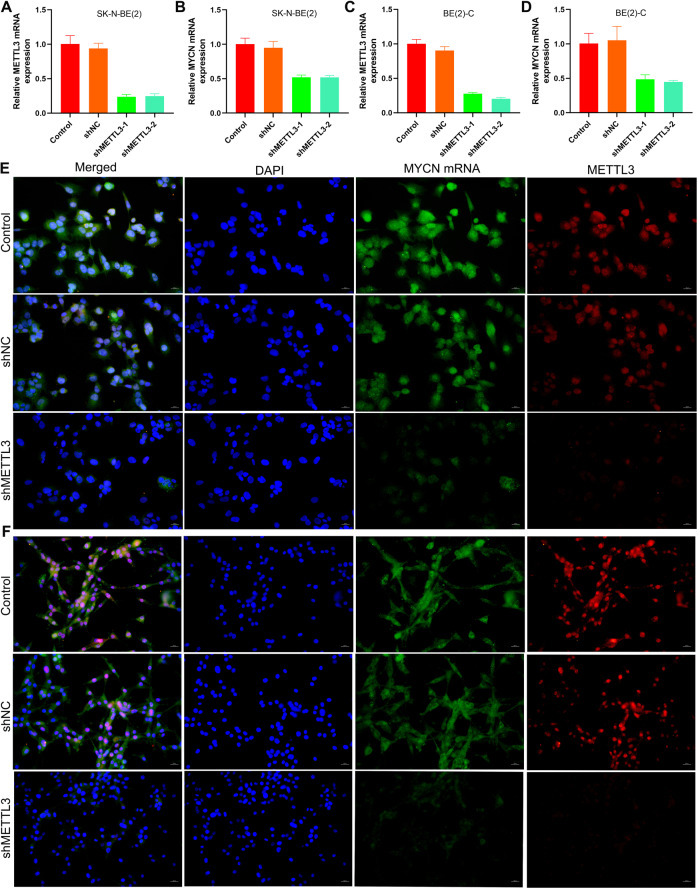
Repression of m6A activation inhibited N-myc expression in MYCN-amplified NB cells. **A** qRT-PCR analysis of the expression level of METTL3 mRNA in SK-N-BE(2) (*n* = 3); **B** qRT-PCR analysis of the expression level of MYCN mRNA in SK-N-BE(2) (*n* = 3); **C** qRT-PCR analysis of the expression level of METTL3 mRNA in BE(2)-C (*n* = 3); **D** qRT-PCR analysis of the expression level of MYCN mRNA in BE(2)-C (*n* = 3); **E** RNA-FISH and immunofluorescence analysis of the expression level of MYCN mRNA and METTL3 in SK-N-BE(2) (Green: MYCN mRNA; Red: METTL3 protein; Blue: DAPI); **F** RNA-FISH and immunofluorescence analysis of the expression level of MYCN mRNA and METTL3 in BE(2)-C (Green: MYCN mRNA; Red: METTL3 protein; Blue: DAPI).

As IGF2BP3 is an important “reader” in m6A RNA methylation regulation, it may affect N-myc expression through the m6A RNA methylation pathway. Therefore, the level of m6A RNA methylation in cells was reduced by downregulating the expression of METTL3 (Fig S2A, B) to further observe whether the expression level of N-myc and IGF2BP3 was affected. This paper found that the expression level of MYCN (Fig. 5B, D–F), N-myc, and IGF2BP3 (Supplementary Fig. S2C, D) was decreased, and the clonability of cells was also decreased (Supplementary Fig. S3) after we reduced the level of m6A by knockdown of the methyltransferase METTL3.

### IGF2BP3 regulated MYCN RNA stability via reading m6A modification

**Fig. 6:**
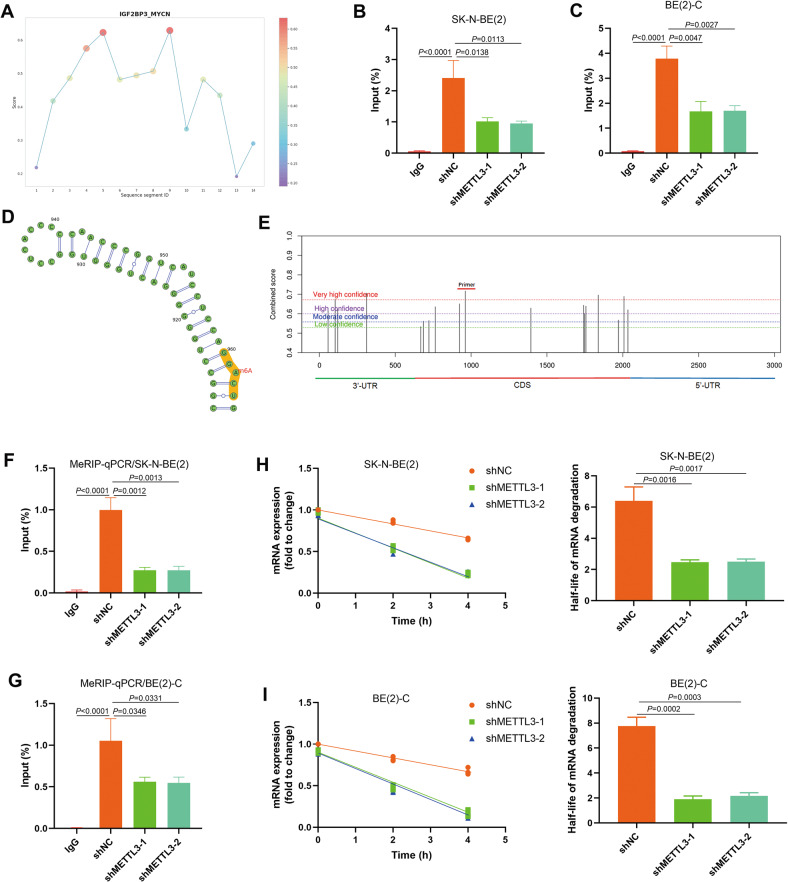
IGF2BP3 regulated NB cell proliferation via reading m6A modification of MYCN. **A** IGF2BP3 could bind to MYCN RNA predicted by RBPsuite website tools; **B** The enrichment of IGF2BP3 in the mRNA of MYCN performed by RIP-qPCR assay in SK-N-BE(2) (*n* = 3); **C** The enrichment of IGF2BP3 in the mRNA of MYCN performed by RIP-qPCR assay in BE(2)-C (*n* = 3); **D**, **E** The m6A modification site of MYCN predicted by SRAMP website tools based on sequence-derived features, and primers designed for MeRIP-qPCR assay. **F** Obvious m6A modification of MYCN confirmed by MeRIP-qPCR, and knockdown of m6A writer METTL3 repressed MYCN m6A modification in SK-N-BE(2) (*n* = 3); **G** Obvious m6A modification of MYCN confirmed by MeRIP-qPCR, and knockdown of m6A writer METTL3 repressed MYCN m6A modification in BE(2)-C (*n* = 3); **H** The mRNA stability and degradation halftime of MYCN in SK-N-BE(2) treated by Actinomycin D (*n* = 3); **I** The mRNA stability and degradation halftime of MYCN in BE(2)-C treated by Actinomycin D (*n* = 3).

Since IGF2BP3 may regulate the expression of MYCN RNA through the m6A pathway, we further analyzed whether the two directly combine. IGF2BP3 was shown to bind to MYCN RNA using RBPsuite analysis (Fig. 6A). RIP-qPCR experiments further validated that IGF2BP3 could bind to MYCN RNA in NB cells (Fig. 6B, C). M6A modification sites of MYCN were revealed by SRAMP website analysis, and the highest confidence region was selected to design qPCR primers for meRIP-qPCR detection (Fig. 6D, E). The results indicated the presence of m6A modification of MYCN RNA in both SK-N-BE(2) and BE(2)-C cells (Fig. 6F, G). Upon downregulation of cellular m6A levels, MYCN RNA degradation was significantly accelerated by actinomycin D treatment, and the RNA degradation half-life was greatly accelerated (Fig. 6H, I).

### N-myc promotes the expression of IGF2BP3

**Fig. 7:**
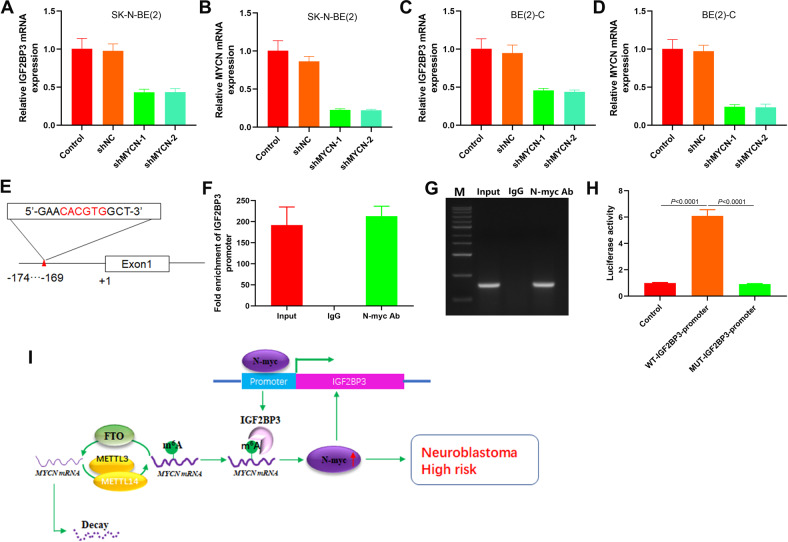
N-myc directly promotes the IGF2BP3 expression in NB cells. **A** qRT-PCR analysis of the expression level of IGF2BP3 mRNA in SK-N-BE(2) (*n* = 3); **B** qRT-PCR analysis of the expression level of MYCN mRNA in SK-N-BE(2) (*n* = 3); **C** qRT-PCR analysis of the expression level of IGF2BP3 mRNA in BE(2)-C (*n* = 3); **D** qRT-PCR analysis of the expression level of MYCN mRNA in BE(2)-C (*n* = 3); **E** Schematic illustration of E-boxes (CANNTG) element in IGF2BP3 promoter; **F**, **G** Analysis of the effect of N-myc on IGF2BP3 promoter by ChIP-qPCR (*n* = 3); **H** Analysis of the targeting effect of N-myc on IGF2BP3 promoter by luciferase reporter assay (*n* = 3); **I** Schematic diagram of a positive feedback loop between RNA N6-methyladenosine reader IGF2BP3 and MYCN facilitates NB proliferation.

The expression level of IGF2BP3 decreased after the knockdown of MYCN expression in SK-N-BE(2) and BE(2)-C cells (Fig. 7A–D). N-myc, as a transcription factor, may regulate IGF2BP3 expression by binding with its promoter. To test this hypothesis, we analyzed the IGF2BP3 promoter region on the Jaspar website and found a possible binding site for N-myc (Fig. 7E). According to the ChIP-qPCR results, the N-myc antibody enriched the DNA of IGF2BP3 promoter region (Fig. 7F, G). The N-myc could activate the promoter activity of wild-type (CACGTG) IGF2BP3 but had no obvious relation with the promoter activity of mutant (AAAAAA) IGF2BP3 (Fig. 7H). These results indicate that N-myc is a transcription factor that directly promotes IGF2BP3 expression in NB cells.

### Overexpression of MYCN in NB cells with IGF2BP3 knockdown partially restored proliferative capacity

Transfection of an MYCN expression plasmid into cells with downregulated IGF2BP3 expression revealed that IGF2BP3 expression levels were partially restored (Supplementary Fig. S4). The clone formation test showed that the upregulation of N-myc expression and cell clonogenic capacity was partially restored (Supplementary Fig. S5A, B, E, F). Nude mouse tumorigenesis assays also showed that upregulation of N-myc expression restored the in vivo proliferation ability of the NB cells (Supplementary Fig. S5C, D, G, H).

## Discussion

Previous studies have shown that MYCN amplification is the main poor prognostic marker of NB, which generally indicates cancer cell metastasis [26]. The study found that the condition of patients with NB would worsen significantly when the level of oncogene expression was increased. However, due to various factors, the academic community has not yet been very clear about the regulation mechanism of MYCN expression. Using cell culture experiments, the result showed that reduced expression of IGF2BP3 contributes significantly to the inhibited proliferation of MYCN-amplified NB cells. The structure of IGF2BP3 is very complex. Research has found that it contains two N-terminal RNA recognition motifs (RRMs) and four C-terminal KH domains [27]. The C-terminal KH domain plays an important role in RNA binding. Some scholars have found that IGF2BP3 plays an important role in the tumorigenicity and pathological progress of many cancers, and its expression level in tumor tissue has significantly increased, so it has high application value in disease diagnosis and prognosis research and can be used as an important biomarker. At present, there are many researches related to this, and a series of achievements have been made [28]. In glioblastoma, IGF2PB3 may promote tumor growth by increasing the secretion of IGF2 [29]. Meanwhile, IGF2BP3 enhances cell proliferation by synergizing with hnRNPM, resulting in enhanced cyclins [30]. Nowadays, IGF2BPs have been found to be members of the m6A reader family. IGF2BP3 can combine with target mRNA in the m6A-dependent mode under specific conditions and has a certain role in promoting the stability of mRNA, improving its translation level [31]. KH domain can bind to m6A RNA. Related experimental studies found that KH3-4 can directly affect the stability of RNA and has a certain correlation with its expression. IGF2BP3 plays a variety of regulatory roles in the biological signal pathway, which is closely related to gene expression and mRNA methylation. At the same time, IGF2BP3 can also promote mRNA splicing and translation and regulate the process of miRNA processing [32].

Few studies have investigated the function of m6A RNA methylation regulation, especially that of IGF2BP3, in NB. This study analyzed the 16 m6A level-regulated enzymes using the GEO and TARGET databases. These enzymes showed differential expression, indicating an active m6A modification in NB. IGF2BP3 is expressed at higher levels in high-risk patients. Univariate Cox regression analysis showed that IGF2BP3 positive coefficient was a risk factor for poor prognosis. IGF2BP3 is also overexpressed and is related to worse prognosis in different tumors, such as gastric carcinogenesis. Moreover, this paper found that the levels of IGF2BP3 and N-myc are positively correlated in NB. IGF2BP3 has a high level in MYCN-amplified NB clinical samples and cell lines.

We aimed to demonstrate the possible molecular mechanism of N-myc regulation by IGF2BP3 in NB. We knocked down IGF2BP3 expression in MYCN-amplified NB cells, and its ability to inhibit cell proliferation was demonstrated using in vivo and in vitro tests. Interestingly, IGF2BP3 knockdown also suppressed N-myc expression. Next, we explored whether IGF2BP3 regulated N-myc expression in an m6A methylation-dependent manner. Using shRNA to knock down METTL3 expression, we found that the N-myc level decreased, while the cell clonability and proliferation capacity were also reduced. The results of RIP-qPCR and MeRIP-qPCR showed that there was strongly m6A-modified MYCN mRNA in IGF2BP3 enrichment products. IGF2BP3 reads m6A modifications in MYCN mRNA, blocking its degradation. Furthermore, we confirmed that MYCN overexpression rescued the inhibition of cell proliferation in IGF2BP3 lower-level cells, as demonstrated by the results of the clone formation test and subcutaneous xenografts of nude mice. So, it can be inferred that IGF2BP3 can promote NB cell proliferation by m6A modification of MYCN.

In addition, we observed that the overexpression of N-myc partially rescued IGF2BP3 expression, and MYCN knockdown also suppressed IGF2BP3 expression. This reflected that N-myc may be associated with the IGF2BP3 expression regulation in NB. N-myc is a transcription factor that forms a complex with the helix-loop-helix leucine zipper protein Max [33]. Therefore, N-myc directly regulates the target genes level that contributes to cell proliferation. A typical E-box (CACGTG) is present in the promoter region of IGF2BP3 (−174 to −169). ChIP and luciferase reporter assays confirmed that N-myc was able to bind to the IGF2BP3 promoter E-box to regulate its expression. These results suggest that in the NBs, IGF2PB3-dependent reading m6A modification of MYCN promotes mRNA stability and that N-myc is also able to upregulate IGF2BP3 expression by binding to its promoter.

In total, this paper verified that the m6A reader IGF2BP3 regulates the proliferation of NB based on the m6A modification of MYCN. N-myc also acts as a transcription factor that regulates IGF2BP3 expression. A positive feedback loop between the RNA N6-methyladenosine reader IGF2BP3 and N-myc facilitates NB cell proliferation (Fig. 7I). This study provides a potential therapeutic target for NB.

## Materials and methods

### GEO datasets and m6A regulators

Expression data and clinical data were retrieved from the GEO and TARGET databases. The RNA sequencing dataset GSE49711 of 498 NB patients was downloaded. The m6A methylation regulators were identified in previous studies [34–37]. To investigate the function of m6A methylation regulators in NB progression, we used the R software to analyze their differential expression in every clinicopathological feature. The prognostic value of m6A modulators was determined by univariate Cox regression analysis using RNA sequencing datasets from 120 NB patients from the TARGET. The correlation between IGF2BP3 and MYCN expression levels in patients with NB was analyzed using linear regression. *P* < 0.05 means the result has statistical significance.

### NB cell lines and primary NB samples

Human NB cell lines with MYCN amplification [SK-N-BE(2), IMR-32, BE(2)-C] and non-MYCN amplification (SK-N-SH, SY-5Y) were cultured at 37 °C in RPMI 1640 medium supplemented with 10% FBS (GIBCO, Grand Island, NY). All cell lines were identified by STR profiling, and the mycoplasma test was negative. Thirty-five paraffin-embedded tissues of NBs diagnosed between 2015 and 2019 at the Shanghai Children’s Hospital, School of Medicine, Shanghai Jiao Tong University, were retrieved. MYCN amplification in the samples was determined by fluorescence in situ hybridization (FISH). All participating patients gave their written informed consent. This study was approved by the Ethical Committee of Shanghai Children’s Hospital, affiliated with Shanghai Jiao Tong University School of Medicine.

### Immunohistochemistry

The paraffin-embedded sections were used to carry out immunohistochemical experiments. After the prepared tissue sections were transparent and dewaxed, all sections were microwave treated in EDTA solution. Then the sections were put into 5% bovine serum albumin for sealing and incubated with anti-IGF2BP3 rabbit polyclonal antibody (1:200; Cat No.14642-1-AP, Proteintech Group. Inc.) for 12 h. Then the sections were treated with HRP combined with rabbit secondary antibody (1:500; Tech Group, Inc.) for 60 min at 23 °C, followed by 3,3′-diaminobenzidine development (Beyotime Biotechnology, Shanghai, China) and hematoxylin staining. The sections were sealed, and images were collected based on a microscope (Nikon, Japan). Expression of IGF2BP3 was determined by cytoplasmic staining intensity using ImageJ software [38].

### shRNA and overexpression plasmid transfection

The shRNAs for human IGF2BP3, METTL3, and MYCN were commercially available from Gene Pharma (Shanghai, China). The MYCN overexpression plasmid, pex-3-MYCN, was commercially available from Gene Pharma. Lipofectamine 3000 transfection reagent (Thermo Fisher Scientific, Waltham, MA, USA) was applied for all the transfection assays. The control group are non-transfected cells, shNC are cells transfected with an shRNA control, and shNC+vector are cells transfected with an shRNA control and empty plasmid.

### RNA extraction and qRT-PCR testing

Cultured cells were harvested to extract total RNA using the TRIzol reagent (Thermo Fisher Scientific). The cDNA synthesis was carried out based on the Hifair II 1st Strand cDNA Synthesis Kit (Yeasen Biotechnology, Shanghai, China). The variations in mRNA expression of related genes were quantified by qRT-PCR, which was performed in triplicate using Hieff® qPCRMix (Yeasen Inc Ltd) and the Heal Force Real-Time System (Heal Force, Shanghai, China). The incubate condition was as follows: 95 °C for 30 s, followed by 40 cycles at 95 °C for 8 s and 60 °C for 30 s. The primers are shown in Supplementary Table S1. The 2^−ΔΔCt^ method was used to determine the results.

### Protein extraction and western blot analysis

Total cellular proteins were extracted from each group using a cell lysis solution (Sigma-Aldrich, St. Louis, MO, USA). Equal amounts (20 μg) of protein were determined using a BCA protein assay kit (Thermo Fisher Scientific) and separated on 10% SDS-PAGE gels. Proteins were then transferred to PVDF membranes (0.45 um, Amersham Biosciences, Piscataway, NJ, USA). The membranes were blocked for 2 h at 37 °C with 5% non-fat milk in Tris Buffered saline with Tween 20 (TBST) and then incubated with anti-IGF2BP3 (1:1000, Cat No.14642-1-AP, Proteintech Group. Inc.), METTL3 (1:1000, Cat No. 15073-1-AP, Proteintech Group. Inc), and N-myc (1:1000, Cell Signaling Technology, Boston, MA, USA) rabbit polyclonal antibodies at 4 °C for 12 h. Anti-β-actin rabbit polyclonal antibody (1:4000, Proteintech Group. Inc.) was used as the loading control and normalization. The secondary antibodies were anti-rabbit IgG conjugated to horseradish peroxidase (HRP) (Proteintech Group. Inc). The secondary antibodies were used at a 1:4000 dilution and were incubated for 1 h at 37 °C. The bands were visualized with ECL reagents (Thermo Fisher Scientific, Waltham, MA, USA) and developed using Tanon 5200 (Tanon, Shanghai, China).

### RNA-FISH and immunofluorescence

Digoxigenin-labeled RNA probes were purchased from Gene Pharma (Shanghai, China). The FISH assay was performed following a previously published protocol [39]. Briefly, the cells were fixed in 4% paraformaldehyde, permeabilized in a 1:1 solution of acetone and methanol, and hybridized with digoxigenin-labeled MYCN sense or antisense RNA probes. After peroxidase quenching and blocking, the hybridized sections were incubated with peroxidase-conjugated anti-digoxigenin antibody (Roche, Indianapolis, IN, USA) and visualized using SuperGloTM Green Immunofluorescence Amplification Kits (Fluorescent Solutions, Augusta, GA, USA). Subsequently, the corresponding antibodies were used to label IGF2BP3 (1:100, Cat No. 14642-1-AP, Proteintech Group, Inc.) or METTL3 (1:200, Cat No. 15073-1-AP, Proteintech Group, Inc.). Alexa Fluor 555 labeled donkey anti-rabbit IgG (Beyotime Biotechnology) was used for fluorescence detection. Nuclei were counter-stained with DAPI. Images were obtained based on Nikon80i microscope (Nikon, Japan).

### Plate clone formation detection

During this test, 5 × 10^3^ cells were seeded in a 10-cm cell culture dish and incubated at 37 °C. Clone size was observed daily under a microscope until the number of cells in the majority of the clones was >50. The medium was then removed, and the cells were stained with 0.2% crystal violet (Sigma-Aldrich) for 30 min. The cells were washed three times with PBS, photographed, and the clones were counted.

### RIP-qPCR

RIP experiments were performed using the EZMagna RIP kit (Millipore) according to the manufacturer’s instructions. Briefly, the cells were harvested and lysed with RIP lysis buffer. Then the lysates were incubated with 5 μg anti-IGF2BP3 rabbit antibody (1:100, Cat No. 14642-1-AP, Proteintech Group, Inc.) or rabbit IgG (Proteintech Group, Inc.) pre-conjugated protein A/G Magnetic Beads (Millipore, Billerica, MA, USA) in 500 μL IP buffer supplemented with RNase inhibitors at 4 °C overnight. The IP complex was treated with proteinase K for 1 h at 52 °C, and RNA was purified. Then qPCR analysis was carried out to determine the concentration of MYCN RNA. The primers used for qPCR are listed in Supplementary Table S1.

### MeRIP-qPCR

Total RNA was extracted by using RNA Miniprep Systems (Promega, WI, USA), and mRNA was further purified via polyAT tract mRNA Isolation Systems (Promega, WI, USA). Then we select Magna MeRIP m6A kit (Millipore) to conduct m6A RNA immunoprecipitation. IP production amplification is performed by qRT-PCR. The MYCN primers used for qPCR are listed in Supplementary Table S1.

### RNA stability assay

The cells were treated with 5 μg/mL actinomycin D (Med Chem Express, Shanghai, China). After incubation for 0, 2, and 4 h, cells were collected, and RNA was extracted for qRT-PCR as described above. The mRNA degradation rate was estimated according to published protocols [40].

### Subcutaneous xenografts of nude mice

Male 5-week-old BALB/c-nu mice were provided by Shanghai SLAC Laboratory Animal CO. LTD. Cells (5 × 10^6^ cells suspended in 0.1 mL PBS) were injected subcutaneously from the axilla of each nude mouse. After 12 days, the long (L) and short (S) diameters of the tumors were measured with vernier caliper every 3 days (0.5 × L × S^2^). The growth curve of subcutaneous tumors was drawn on the basis of the measured tumor volume. The experimental subjects were killed 33 days after the injection of cells, and subcutaneous tumors were removed completely. All experimental procedures were approved by the Institutional Animal Care and Utilization Committee of Shanghai Children’s Hospital, affiliated with Shanghai Jiao Tong University School of Medicine.

### RNA m6A quantification

Total RNA was extracted using TRIzol (Thermo Fisher Scientific), and its quality was evaluated by Nanodrop (Thermo Fisher Scientific) and compared. According to instructions, the EpiQuik m6A RNA kit (Epigentek, NY, USA) was used to detect the m6A modification level of total RNA. Briefly, 200 ng RNA accompanied with m6A standard was coated on assay wells, followed by the capture antibody solution and detection antibody solution. m6A levels were quantified colorimetrically by reading the absorbance of each well at a wavelength of 450 nm (OD450), and calculations were performed based on the standard curve.

### Chromatin immunoprecipitation (ChIP)-qPCR assay

During the ChIP experiment, we use SimpleChIP according to the relevant instructions ® IP Kit (Epigentek, NY, USA) detection; the corresponding experimental process is as follows. Briefly, the chromatin was segmented by ultrasonic treatment after 1 × 10^7^ cells were cross-linked with formaldehyde and then incubated with anti-human N-myc antibody (Cell Signaling Technology) at 4 °C overnight; histone H3 (D2B12) XP® Rabbit mAb (Cell Signaling Technology) was used as the positive control, and Rabbit mAb IgG XP® Isotype (Cell Signaling Technology) was used as the negative control. ChIP-grade protein G magnetic beads were added and incubated at 4 °C for 2 h, and DNA on the beads was eluted. The standard curve was generated by real-time PCR with continuous diluents (undiluted, 1:5, 1:25, and 1:125) of 2% input chromatin DNA, and the enrichment degree of IGF2BP3 promoter DNA in different groups of samples was determined.

### Luciferase reporter assay

To analyze the regulatory effect of N-myc on the IGF2BP3 promoter, we constructed a wild-type pGL3-Basic-IGF2BP3 (CACGTG) plasmid and mutant pGL3-Basic-IGF2BP3 (AAAAAA) plasmid based on the sequence of responsive elements on the IGF2BP3 promoter. The pex-3-MYCN (Gene Pharma) plasmid expressing N-myc was co-transfected into HEK293 cells with pGL3-Basic-IGF2BP3 (CACGTG), pGL3-Basic-IGF2BP3 (AAAAAA), or pGL3-Basic. After culturing for 48 h, the cells were split using the Dual Luciferase Reporter Assay System (Promega), according to the instructions. The results were analyzed using BioTek Synergy H1 (BioTek, USA) after luminescence was added.

### Statistical analysis

All statistical analysis was performed by SPSS software (version 22.0, IBM Corp., Armonk, NY, USA). GraphPad Prism (version 8.03; GraphPad Software, La Jolla, CA, USA) was used to determine the statistical results. Data are shown as mean ± standard error of the mean (mean ± SD). Statistical analysis of the data from the two groups was performed using Student’s *t*-test. Comparisons of multiple groups were performed by one-way ANOVA followed by an LSD-*t*-test. *P* < 0.05 indicates statistical difference. At least three samples were included in an independent experiment.

## Supplementary information

Supplementary materials

Original Data File

## Data Availability

The datasets used and/or analyzed during the current study are available from the corresponding author upon reasonable request.

## References

[1] Matthay KK, Maris JM, Schleiermacher G, Nakagawara A, Mackall CL, Diller L (2016). Neuroblastoma. Nat Rev Dis Prim.

[2] Lundberg KI, Treis D, Johnsen JI (2022). Neuroblastoma heterogeneity, plasticity, and emerging therapies. Curr Oncol Rep.

[3] Zafar A, Wang W, Liu G, Wang X, Xian W, McKeon F (2021). Molecular targeting therapies for neuroblastoma: progress and challenges. Med Res Rev.

[4] Bouchard C, Staller P, Eilers M (1998). Control of cell proliferation by Myc. Trends Cell Biol.

[5] Yoshida GJ (2018). Emerging roles of Myc in stem cell biology and novel tumor therapies. J Exp Clin Cancer Res.

[6] Mukherjee B, Morgenbesser SD, DePinho RA (1992). Myc family oncoproteins function through a common pathway to transform normal cells in culture: cross-interference by Max and trans-acting dominant mutants. Genes Dev.

[7] Brodeur GM, Seeger RC, Schwab M, Varmus HE, Bishop JM (1984). Amplification of N-myc in untreated human neuroblastomas correlates with advanced disease stage. Science.

[8] Schwab M, Ellison J, Busch M, Rosenau W, Varmus HE, Bishop JM (1984). Enhanced expression of the human gene N-myc consequent to amplification of DNA may contribute to malignant progression of neuroblastoma. Proc Natl Acad Sci USA.

[9] Maris JM (2010). Recent advances in neuroblastoma. N Engl J Med.

[10] Ponzoni M, Bachetti T, Corrias MV, Brignole C, Pastorino F, Calarco E (2022). Recent advances in the developmental origin of neuroblastoma: an overview. J Exp Clin Cancer Res.

[11] Wang T, Kong S, Tao M, Ju S (2020). The potential role of RNA N6-methyladenosine in cancer progression. Mol Cancer.

[12] Zhou Z, Lv J, Yu H, Han J, Yang X, Feng D (2020). Mechanism of RNA modification N6-methyladenosine in human cancer. Mol Cancer.

[13] Bian J, Zhuo Z, Zhu J, Yang Z, Jiao Z, Li Y (2020). Association between METTL3 gene polymorphisms and neuroblastoma susceptibility: a nine-centre case-control study. J Cell Mol Med.

[14] Zhuo Z, Lu H, Zhu J, Hua RX, Li Y, Yang Z (2020). METTL14 gene polymorphisms confer neuroblastoma susceptibility: an eight-center case-control study. Mol Ther Nucleic Acids.

[15] Tang J, Lu H, Yang Z, Li L, Li L, Zhang J (2021). Associations between WTAP gene polymorphisms and neuroblastoma susceptibility in Chinese children. Transl Pediatr.

[16] Liu J, Cheng J, Li L, Li Y, Zhou H, Zhang J (2021). YTHDF1 gene polymorphisms and neuroblastoma susceptibility in Chinese children: an eight-center case-control study. J Cancer.

[17] Li Y, Lu T, Wang J, Zhuo Z, Miao L, Yang Z (2021). YTHDC1 gene polymorphisms and neuroblastoma susceptibility in Chinese children. Aging..

[18] Zeng H, Li M, Liu J, Zhu J, Cheng J, Li Y (2021). *YTHDF2* gene rs3738067 A>G polymorphism decreases neuroblastoma risk in Chinese children: evidence from an eight-center case-control study. Front Med.

[19] Cheng J, Xu L, Deng L, Xue L, Meng Q, Wei F (2020). RNA N(6)-methyladenosine modification is required for miR-98/MYCN axis-mediated inhibition of neuroblastoma progression. Sci Rep.

[20] Wang SH, Chen YL, Hsiao JR, Tsai FY, Jiang SS, Lee AY (2021). Insulin-like growth factor binding protein 3 promotes radiosensitivity of oral squamous cell carcinoma cells via positive feedback on NF-κB/IL-6/ROS signaling. J Exp Clin Cancer Res.

[21] Ho GYF, Zheng SL, Cushman M, Perez-Soler R, Kim M, Xue X (2016). Associations of insulin and IGFBP-3 with lung cancer susceptibility in current smokers. J Natl Cancer Inst.

[22] Dar AA, Majid S, Nosrati M, de Semir D, Federman S, Kashani-Sabet M (2010). Functional modulation of IGF-binding protein-3 expression in melanoma. J Invest Dermatol.

[23] Hang D, He X, Kværner AS, Chan AT, Wu K, Ogino S (2019). Plasma biomarkers of insulin and the insulin-like growth factor axis, and risk of colorectal adenoma and serrated polyp. JNCI Cancer Spectr..

[24] Regel I, Eichenmüller M, Joppien S, Liebl J, Häberle B, Müller-Höcker J (2012). IGFBP3 impedes aggressive growth of pediatric liver cancer and is epigenetically silenced in vascular invasive and metastatic tumors. Mol Cancer.

[25] Liu Q, Jiang J, Zhang X, Zhang M, Fu Y (2021). Comprehensive analysis of IGFBPs as biomarkers in gastric cancer. Front Oncol.

[26] Huang M, Weiss WA (2013). Neuroblastoma and MYCN. Cold Spring Harb Perspect Med.

[27] Bell JL, Wächter K, Mühleck B, Pazaitis N, Köhn M, Lederer M (2013). Insulin-like growth factor 2 mRNA-binding proteins (IGF2BPs): post-transcriptional drivers of cancer progression?. Cell Mol Life Sci.

[28] Findeis-Hosey JJ, Xu H (2011). The use of insulin like-growth factor II messenger RNA binding protein-3 in diagnostic pathology. Hum Pathol.

[29] Suvasini R, Shruti B, Thota B, Shinde SV, Friedmann-Morvinski D, Nawaz Z (2011). Insulin growth factor-2 binding protein 3 (IGF2BP3) is a glioblastoma-specific marker that activates phosphatidylinositol 3-kinase/mitogen-activated protein kinase (PI3K/MAPK) pathways by modulating IGF-2. J Biol Chem.

[30] Rivera Vargas T, Boudoukha S, Simon A, Souidi M, Cuvellier S, Pinna G (2014). Post-transcriptional regulation of cyclins D1, D3 and G1 and proliferation of human cancer cells depend on IMP-3 nuclear localization. Oncogene..

[31] Huang H, Weng H, Sun W, Qin X, Shi H, Wu H (2018). Recognition of RNA N(6)-methyladenosine by IGF2BP proteins enhances mRNA stability and translation. Nat Cell Biol.

[32] Nazarov IB, Bakhmet EI, Tomilin AN (2019). KH-domain poly(C)-binding proteins as versatile regulators of multiple biological processes. Biochemistry..

[33] Wenzel A, Cziepluch C, Hamann U, Schürmann J, Schwab M (1991). The N-Myc oncoprotein is associated in vivo with the phosphoprotein Max(p20/22) in human neuroblastoma cells. EMBO J.

[34] Yang Y, Hsu PJ, Chen YS, Yang YG (2018). Dynamic transcriptomic m(6)A decoration: writers, erasers, readers and functions in RNA metabolism. Cell Res.

[35] Bi Z, Liu Y, Zhao Y, Yao Y, Wu R, Liu Q (2019). A dynamic reversible RNA N(6)-methyladenosine modification: current status and perspectives. J Cell Physiol.

[36] Zhang W, Qian Y, Jia G (2021). The detection and functions of RNA modification m(6)A based on m(6)A writers and erasers. J Biol Chem.

[37] Zhao Y, Shi Y, Shen H, Xie W (2020). m(6)A-binding proteins: the emerging crucial performers in epigenetics. J Hematol Oncol.

[38] Kang W, Tong JH, Chan AW, Lee TL, Lung RW, Leung PP (2011). Yes-associated protein 1 exhibits oncogenic property in gastric cancer and its nuclear accumulation associates with poor prognosis. Clin Cancer Res.

[39] Long J, Badal SS, Ye Z, Wang Y, Ayanga BA, Galvan DL (2016). Long noncoding RNA Tug1 regulates mitochondrial bioenergetics in diabetic nephropathy. J Clin Invest.

[40] Liu J, Eckert MA, Harada BT, Liu SM, Lu Z, Yu K (2018). m(6)A mRNA methylation regulates AKT activity to promote the proliferation and tumorigenicity of endometrial cancer. Nat Cell Biol.

