# Supplementary material for: RNA N6-methyladenosine reader IGF2BP3 interacts with MYCN and facilitates neuroblastoma cell proliferation
Source: Cell Death Discov. 2023 May 8;9:151. doi: 10.1038/s41420-023-01449-3 (PMC10167253; doi:10.1038/s41420-023-01449-3)
Supplement: Supplementary file 1 — Supplementary materials [file 41420_2023_1449_MOESM1_ESM.docx]

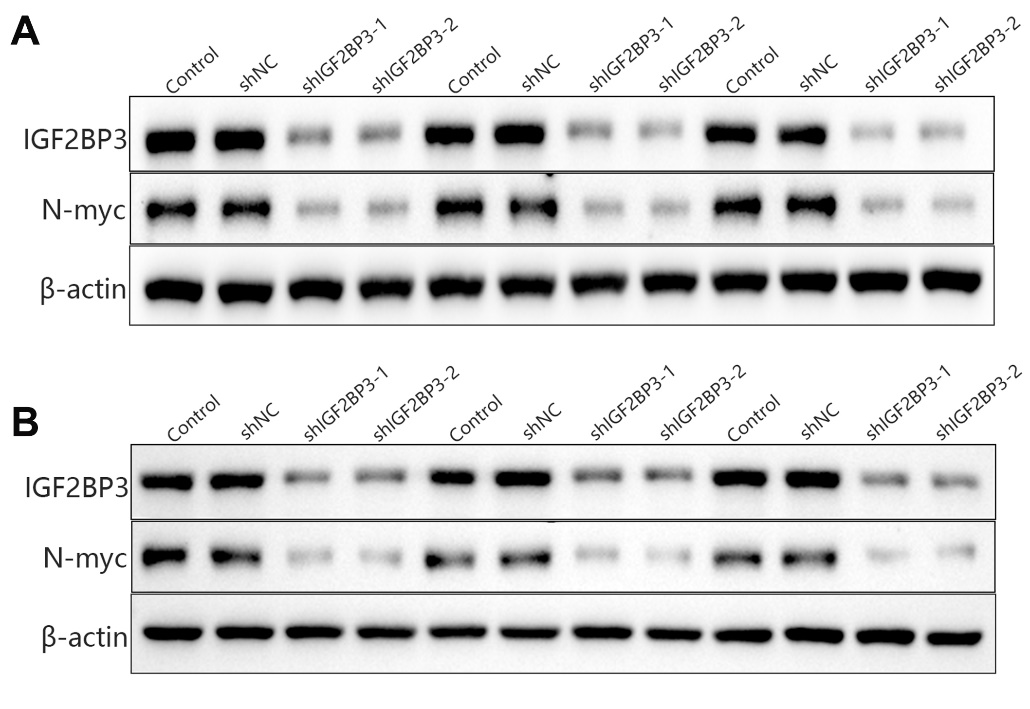


Fig S1. Western blot analysis the expression level of IGF2BP3 and N-myc in SK-N-BE (2) (A) and BE (2)-C (B) (n=3).


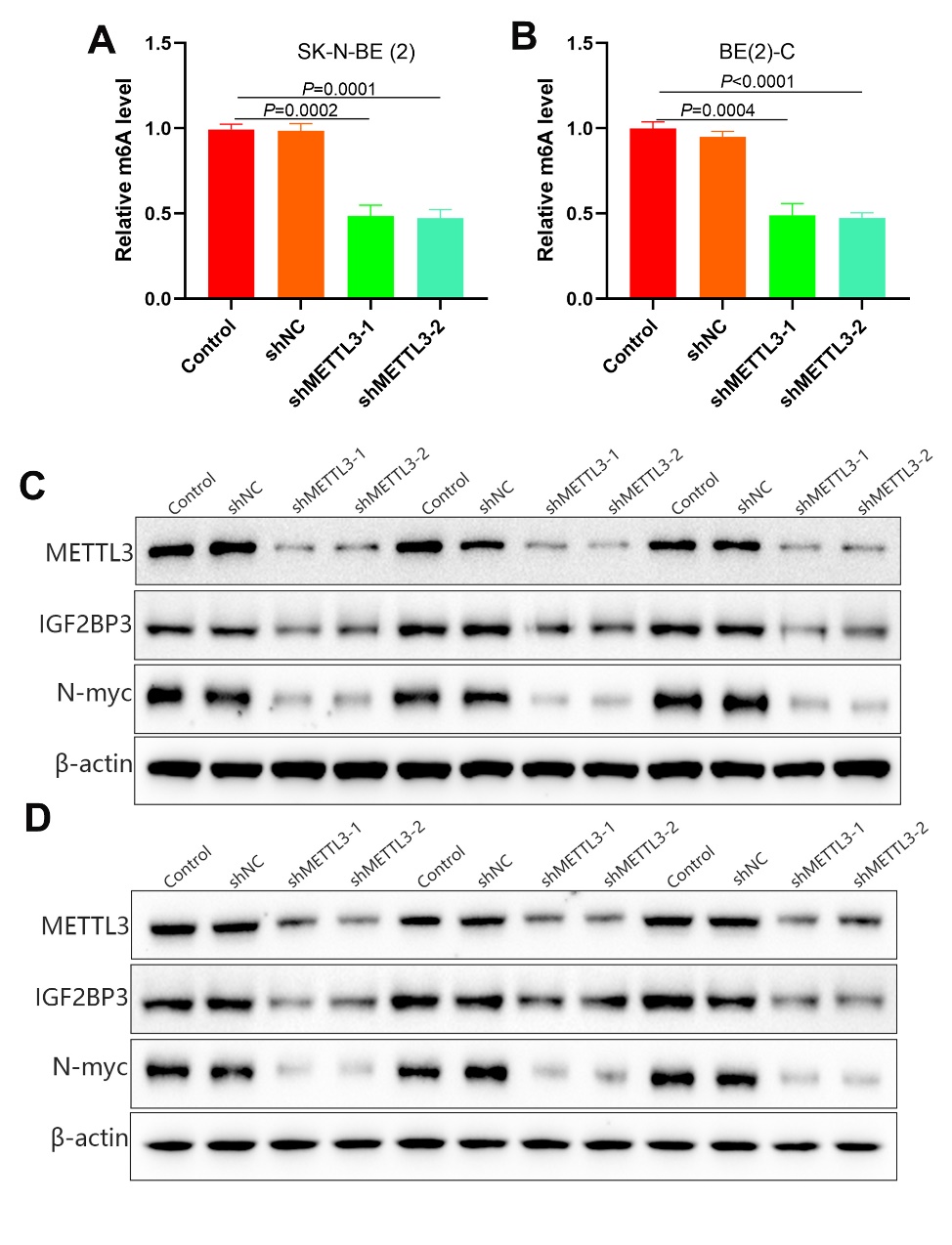


Fig S2. The m6A level and the expression level of METTL3 in SK-N-BE (2) (C) and BE (2)-C (D) (n=3).


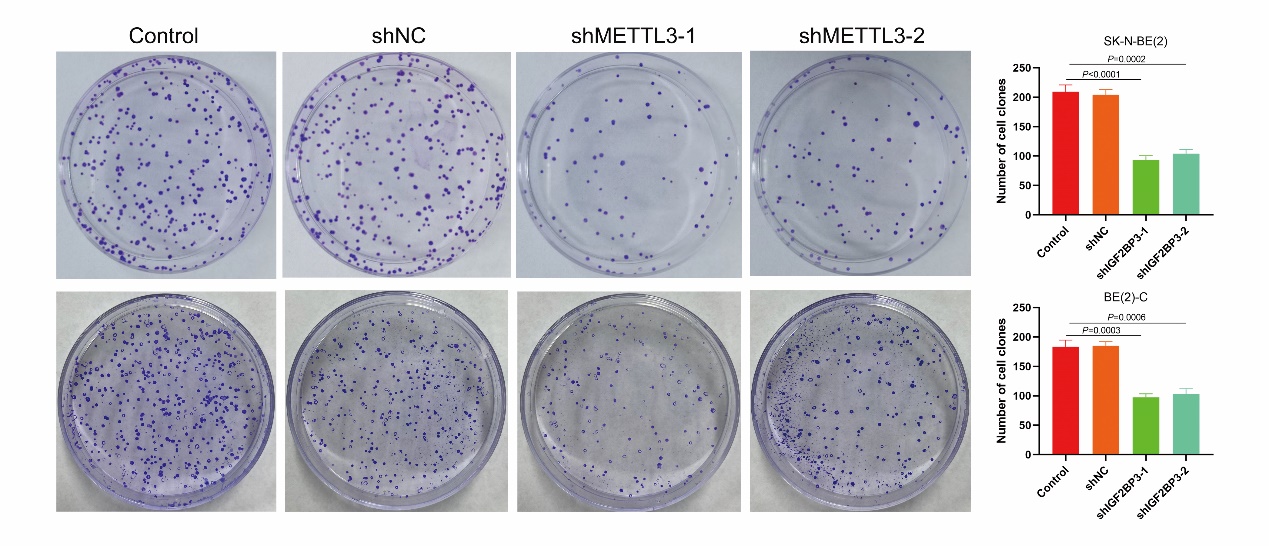


Fig S3. Repression of m6A activation impaired proliferation of SK-N-BE (2) and BE (2)-C (n=3).


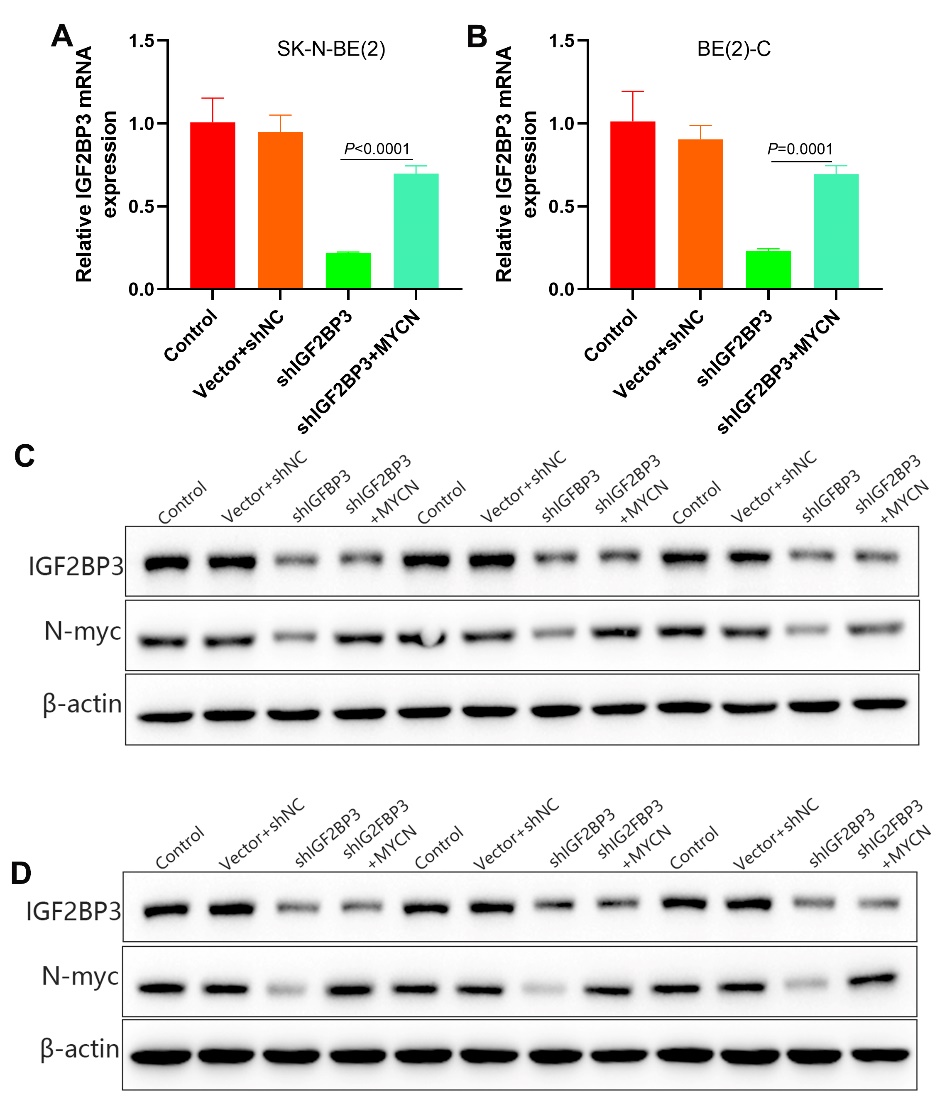


Fig S4. Overexpression of N-myc in NB cells with knockdown of IGF2BP3 was able to partially restore the expression of IGF2BP3

A and B: qRT-PCR analysis the expression level of IGF2BP3 mRNA in SK-N-BE (2) and BE (2)-C (n=3); C and D: Western blot analysis the expression level of IGF2BP3 and N-myc in SK-N-BE (2) (C) and BE (2)-C (D) (n=3).


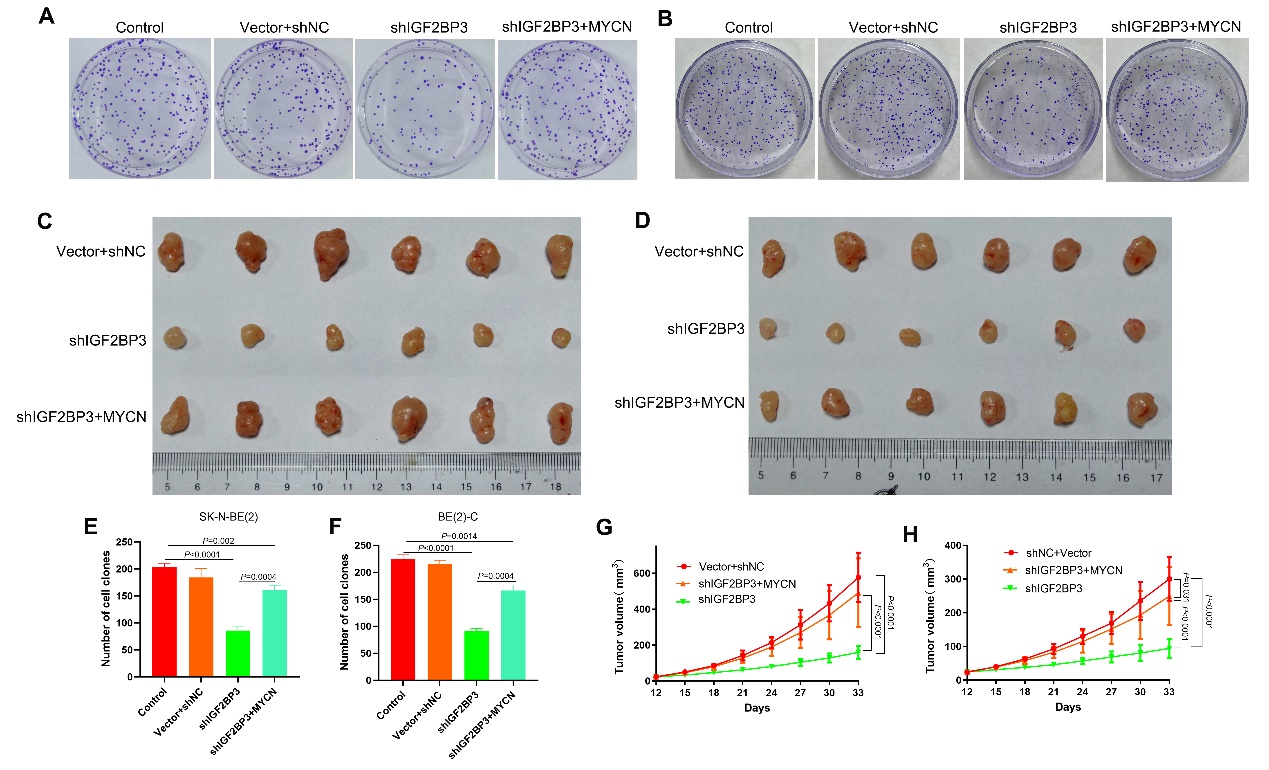


Fig S5. Overexpression of N-myc in NB cells with knockdown of IGF2BP3 was able to partially restore the proliferative capacity. A, B, E and F: Monolayer colonies were detected in SK-N-BE (2) and BE (2)-C (n=3); C and G: Xenograft formation of SK-N-BE (2) cells (n=6); D and H: Xenograft formation of BE (2)-C cells (n=6).

| Table S1 Primers’ sequences used in this study | | |
| --- | --- | --- |
| Gene | forward primer（5’-3’） | reverse primer（5’-3’） |
| IGF2BP3 | TATATCGGAAACCTCAGCGAGA | GGACCGAGTGCTCAACTTCT |
| MYCN | ACCCGGACGAAGATGACTTCT | CAGCTCGTTCTCAAGCAGCAT |
| GAPDH | TGTGGGCATCAATGGATTTGG | ACACCATGTATTCCGGGTCAAT |
| MYCN (RIP-qPCR /MeRIP-qPCR) | CTGTGGGGCAGCCCGGCC | CGCGGCCGTGCTGCAGCTT |
| IGF2BP3 (ChIP-qPCR) | CTCCCAATCTCGTTTCCC | AATCCGCTCCGAGTGTCC |
