# Supplementary material for: RNA N6-methyladenosine reader IGF2BP3 interacts with MYCN and facilitates neuroblastoma cell proliferation
Source: Cell Death Discov. 2023 May 8;9:151. doi: 10.1038/s41420-023-01449-3 (PMC10167253; doi:10.1038/s41420-023-01449-3)
Supplement: Supplementary file 2 — Original Data File [file 41420_2023_1449_MOESM2_ESM.docx]

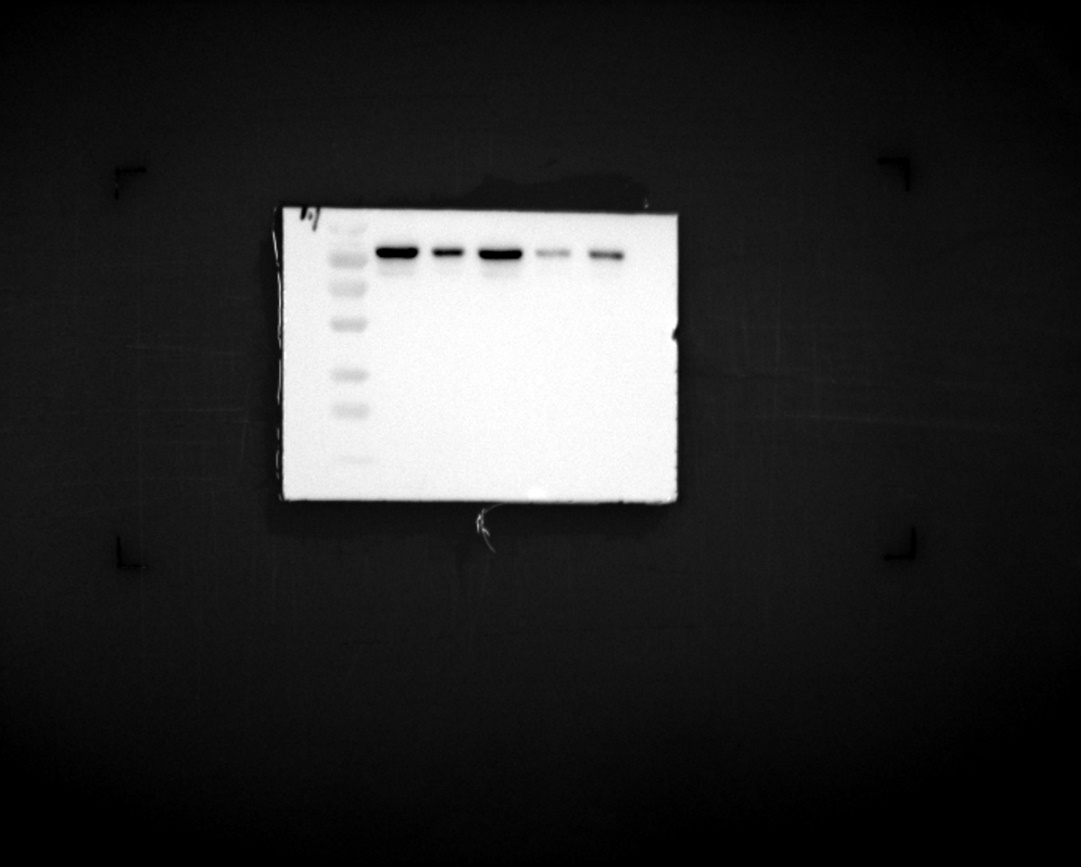


Fig 2-IGF2BP3-1


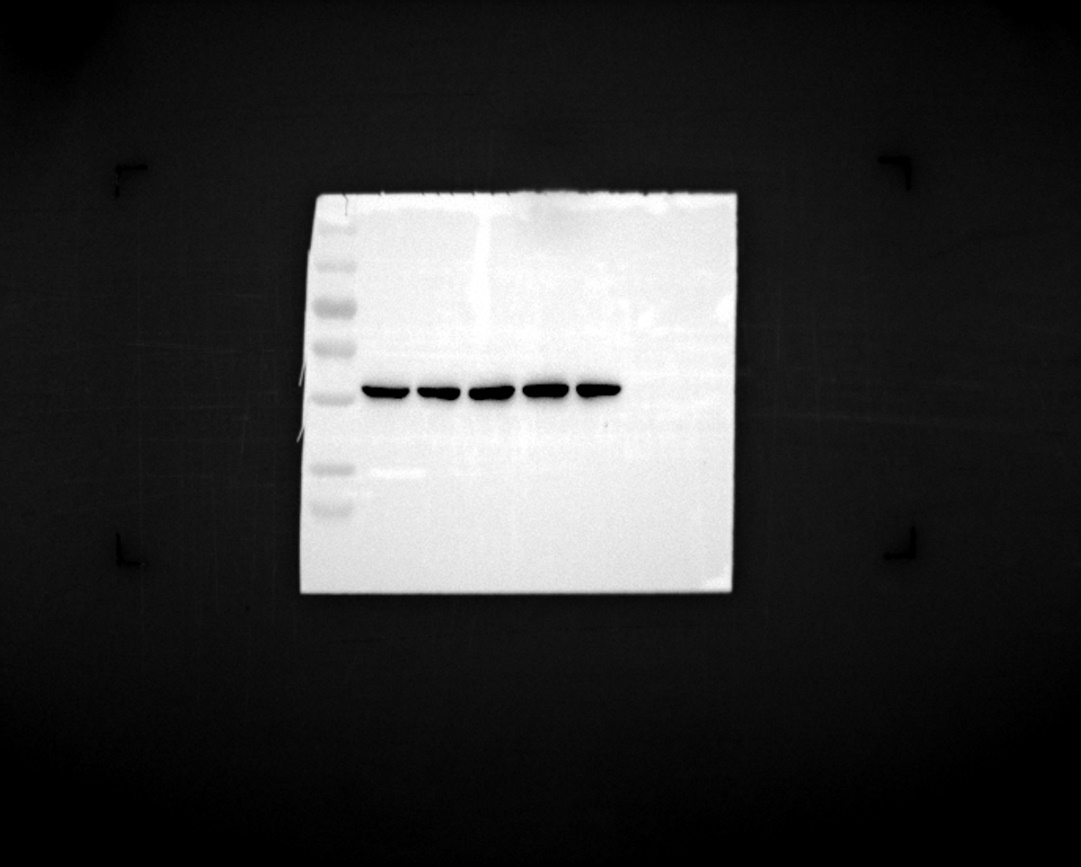


Fig 2-β-actin-1


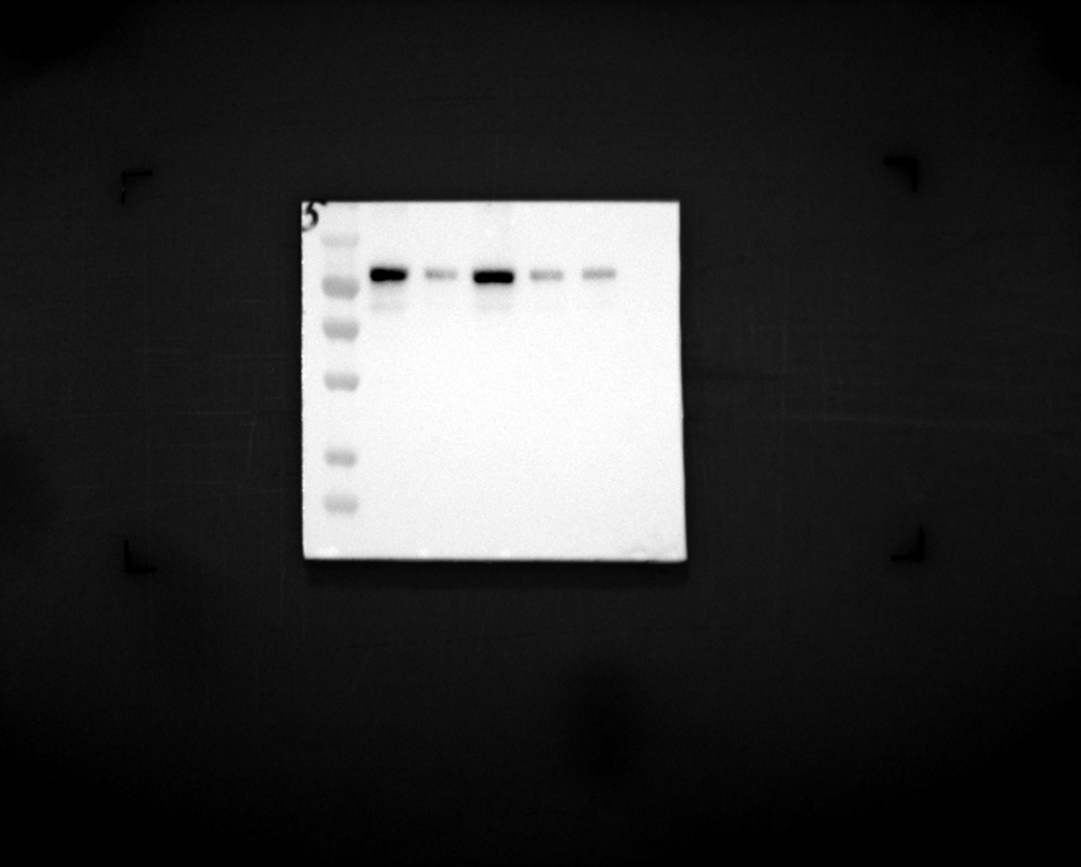


Fig 2-IGF2BP3-2


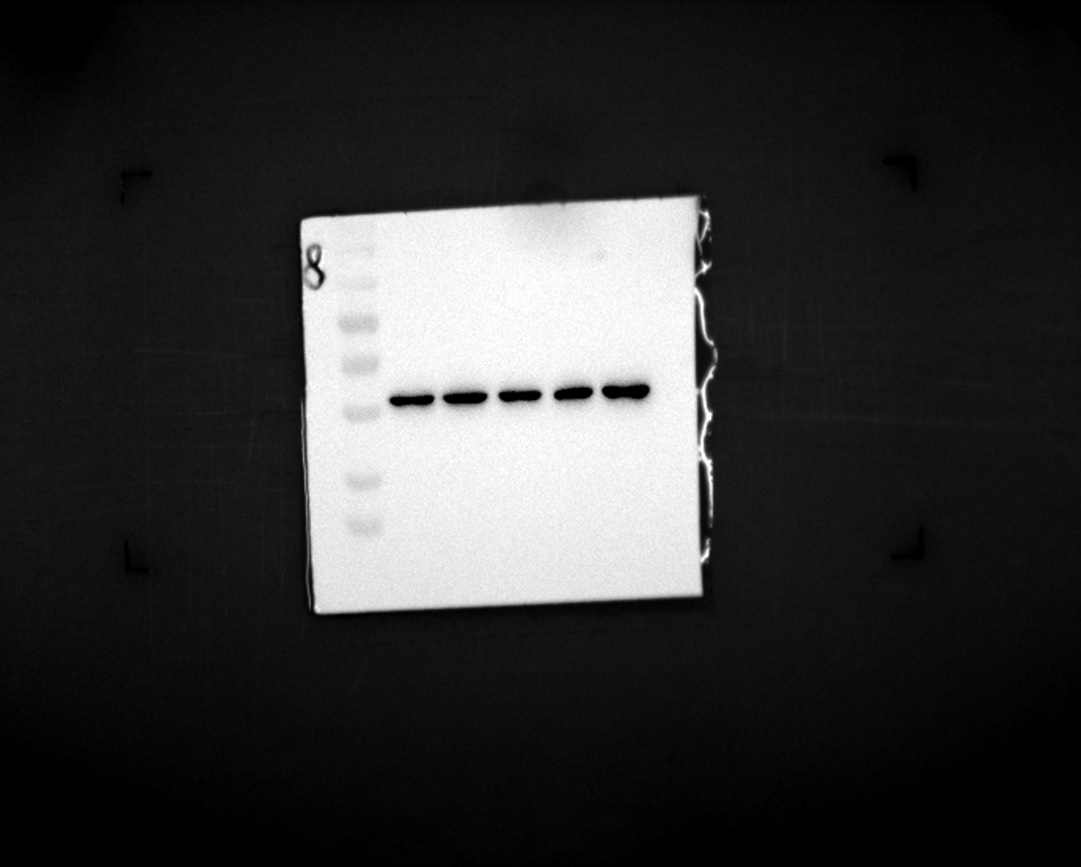


Fig 2-β-actin-2


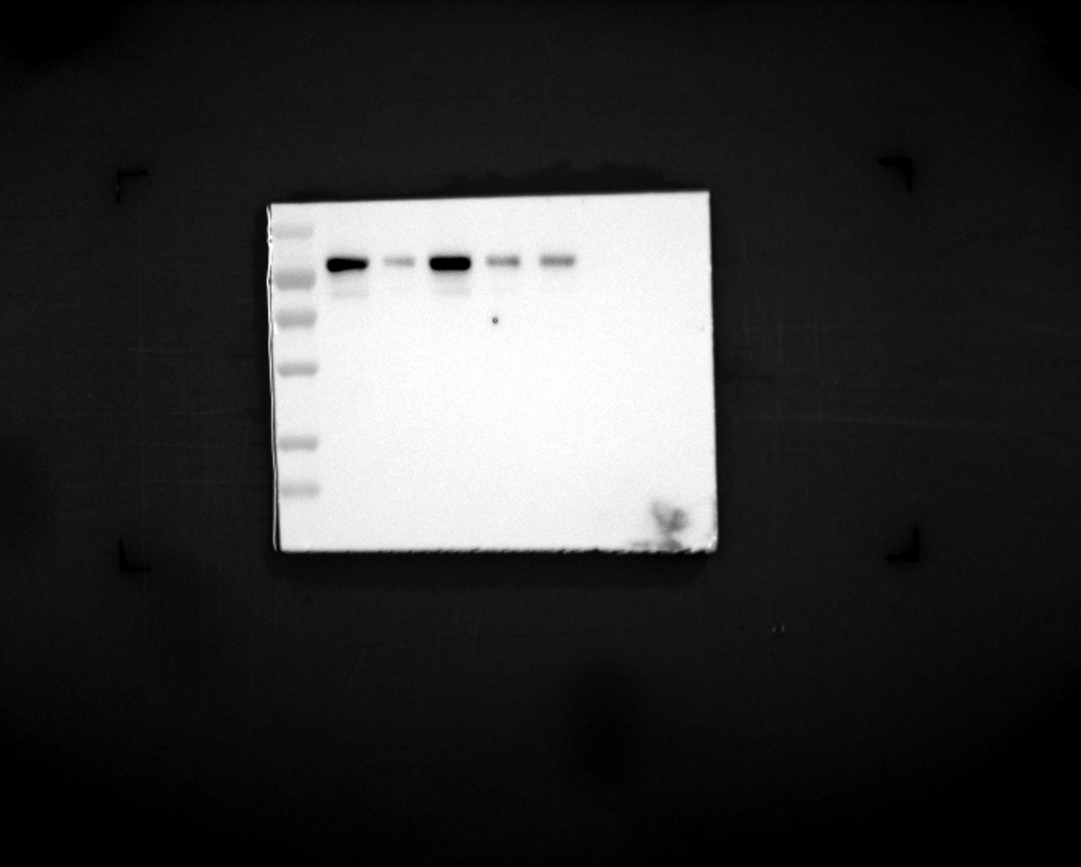


Fig 2-IGF2BP3-3


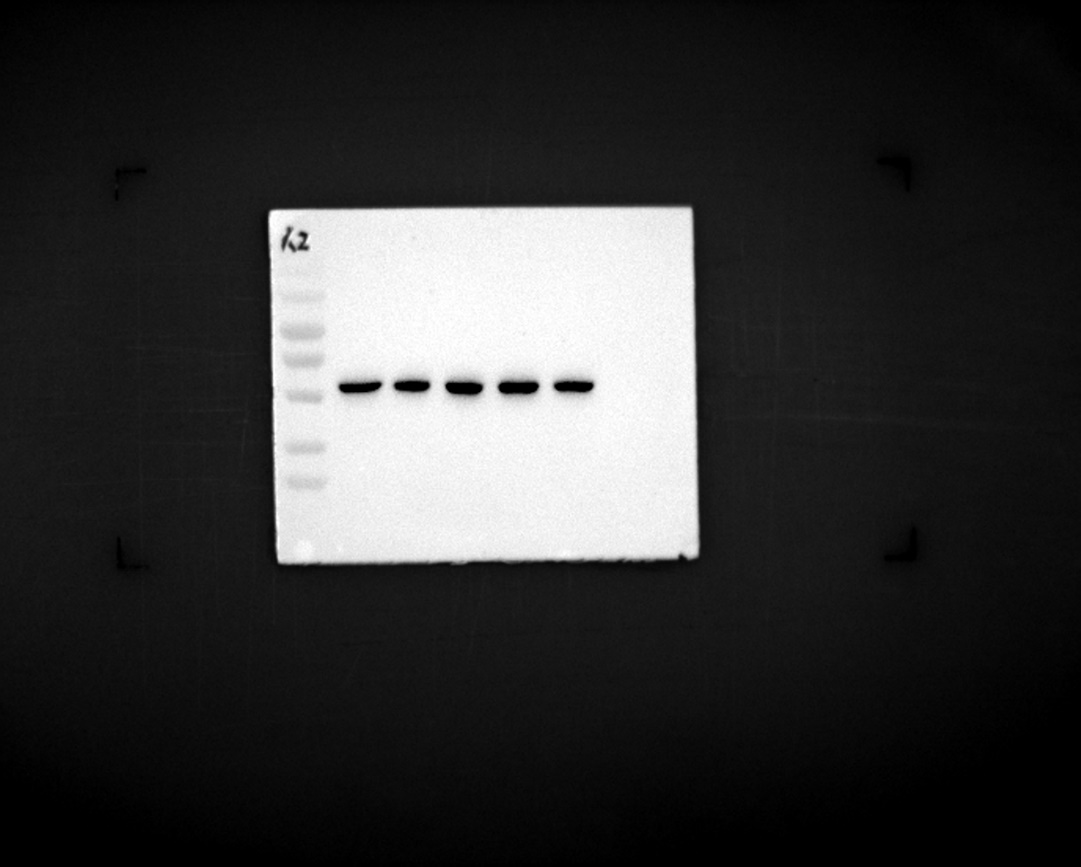


Fig 2-β-actin-3


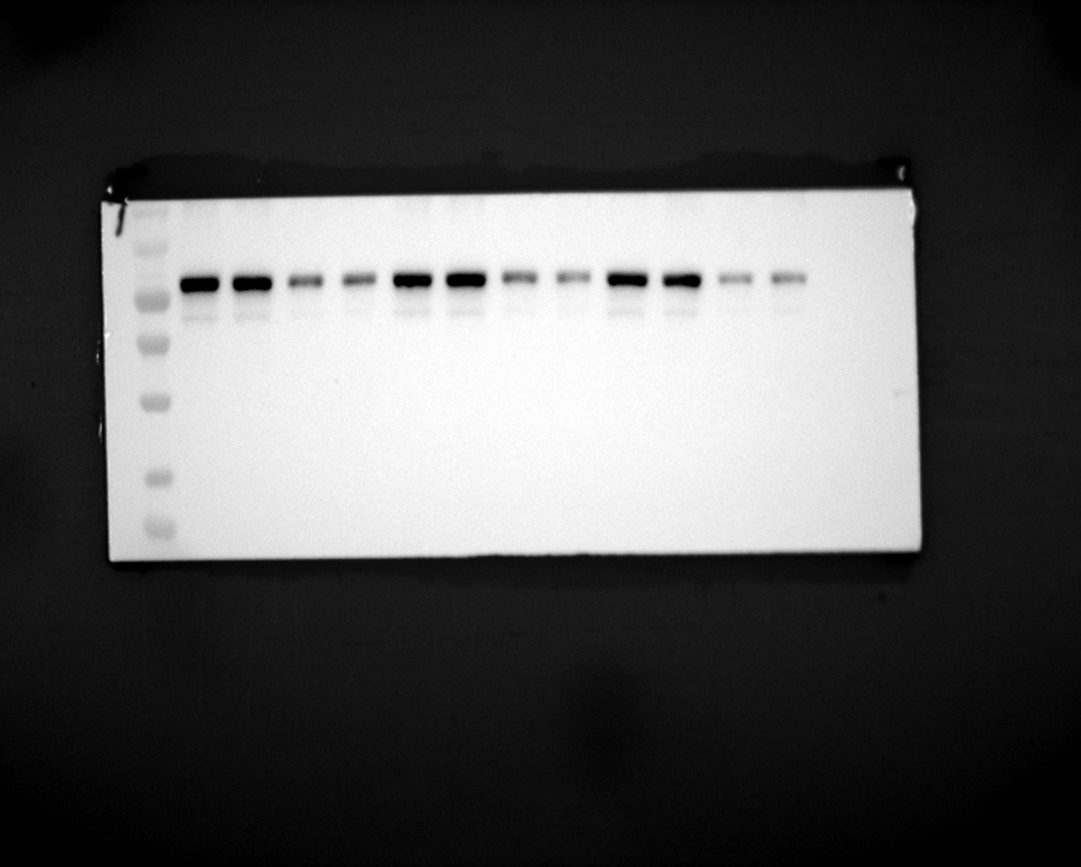


Fig s1-SK-N-BE(2)-IGF2BP3


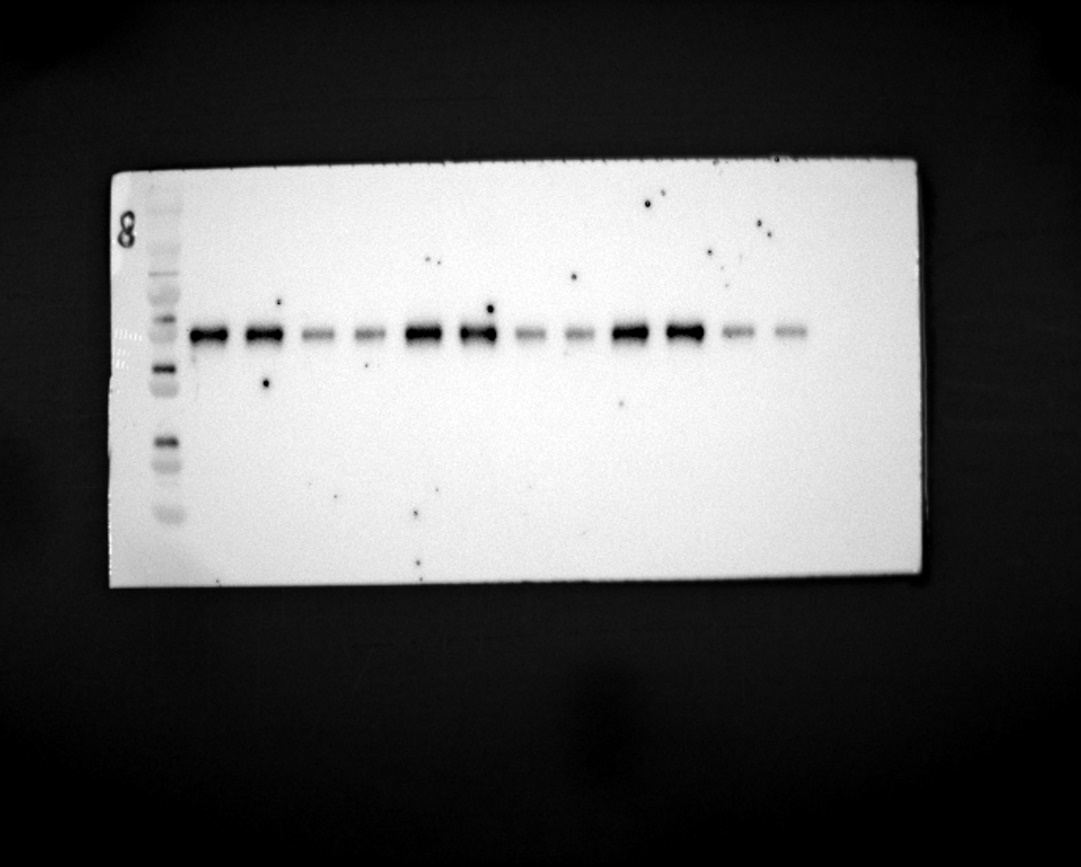


Fig s1-SK-N-BE(2)-N-Myc


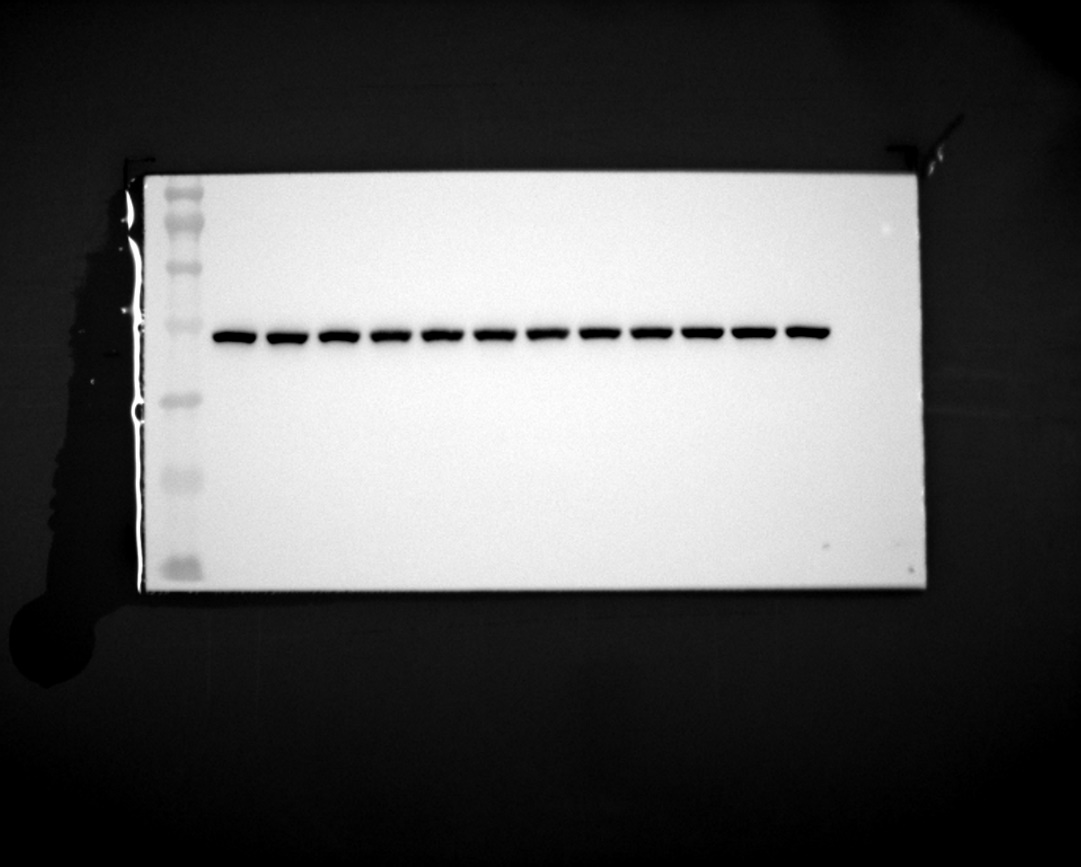


Fig s1-SK-N-BE(2)-β-actin


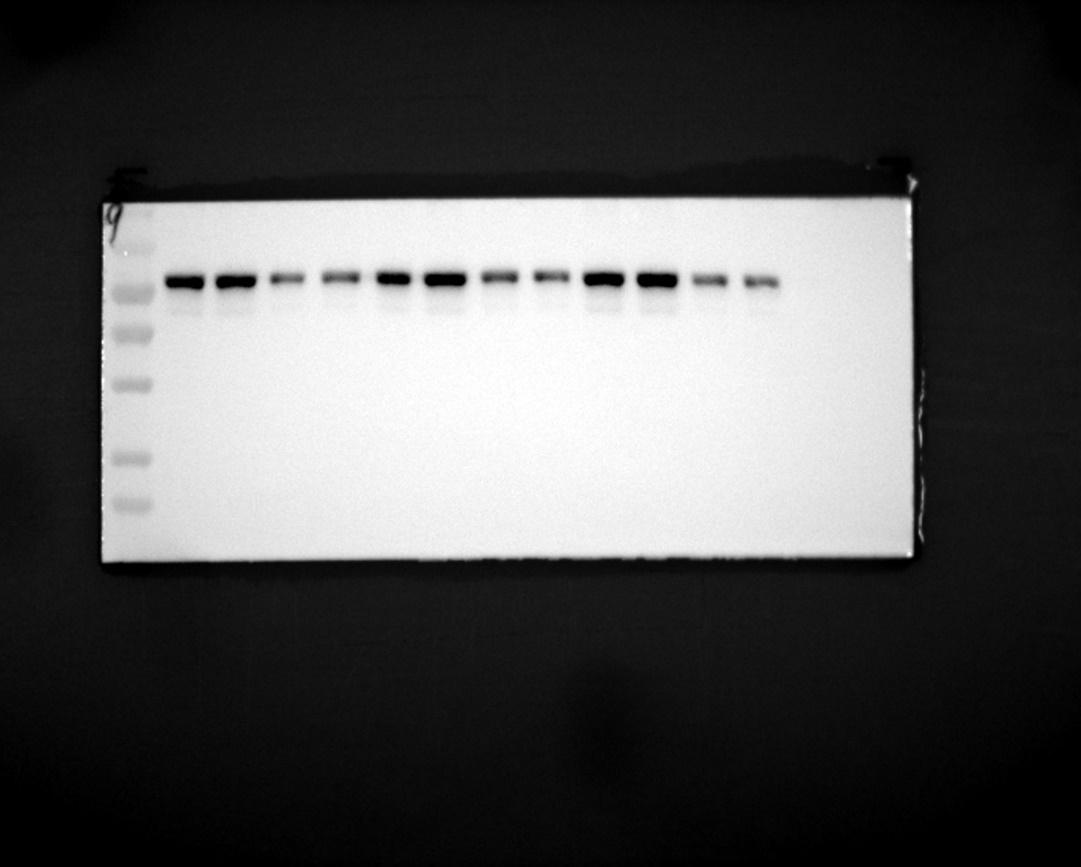


Fig s1-BE(2)-C-IGF2BP3


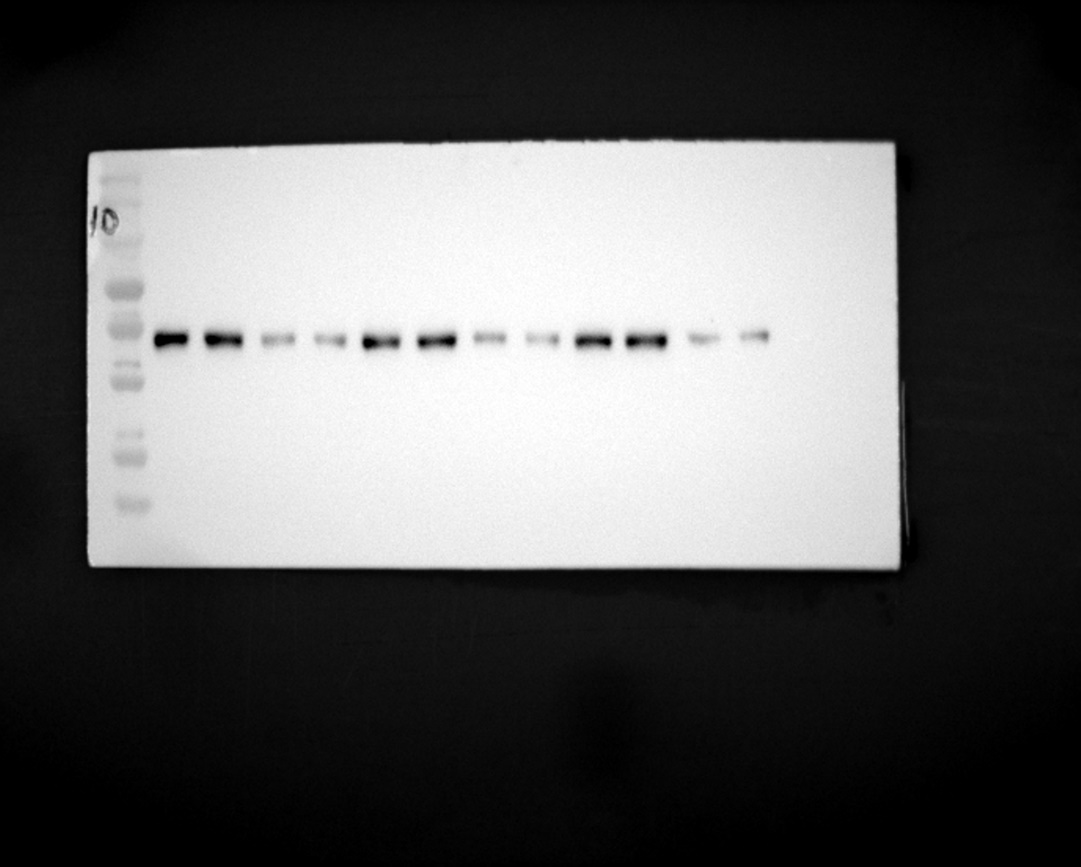


Fig s1-BE(2)-C-N-Myc


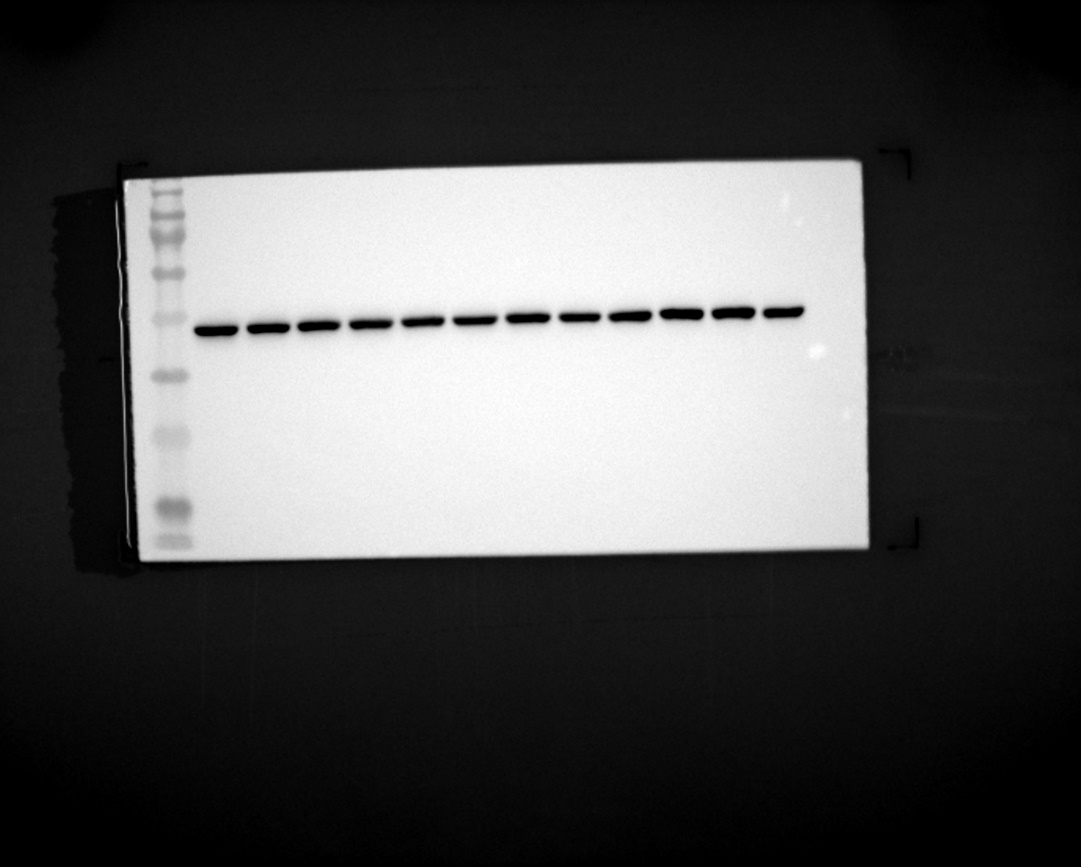


Fig s1-BE(2)-C-β-actin


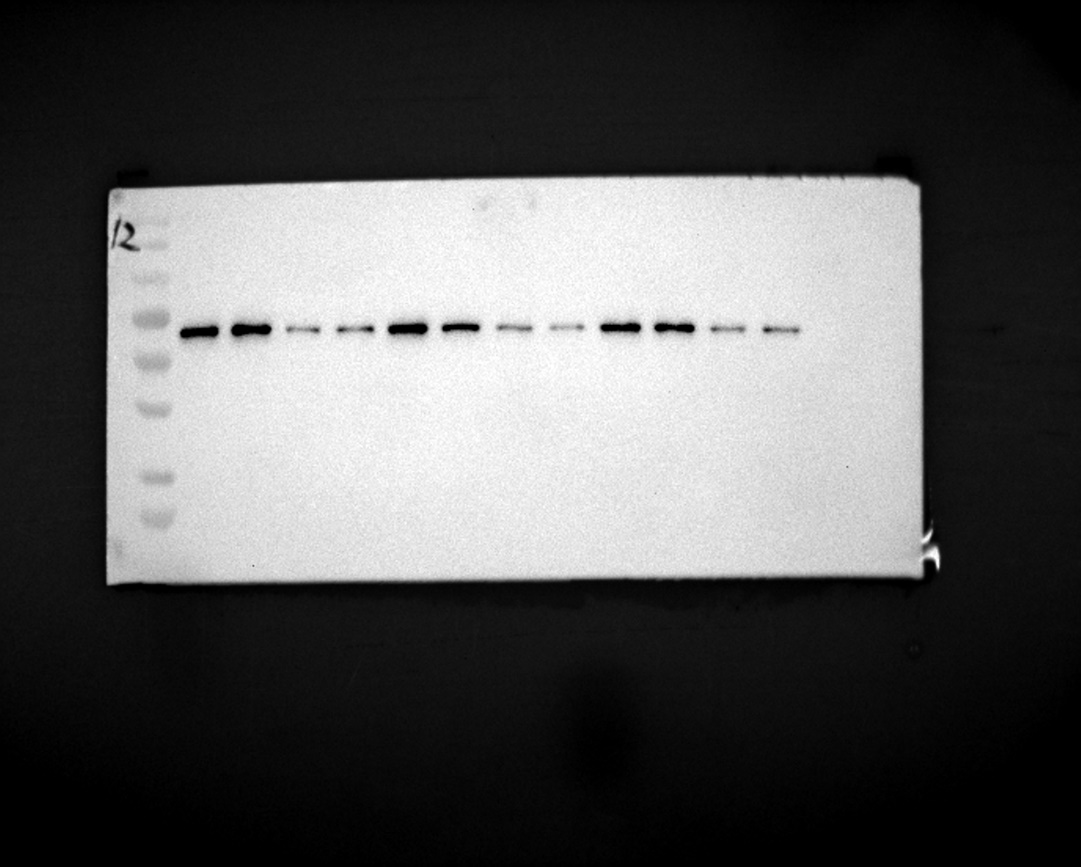


Fig s2-SK-N-BE(2)-METTL3


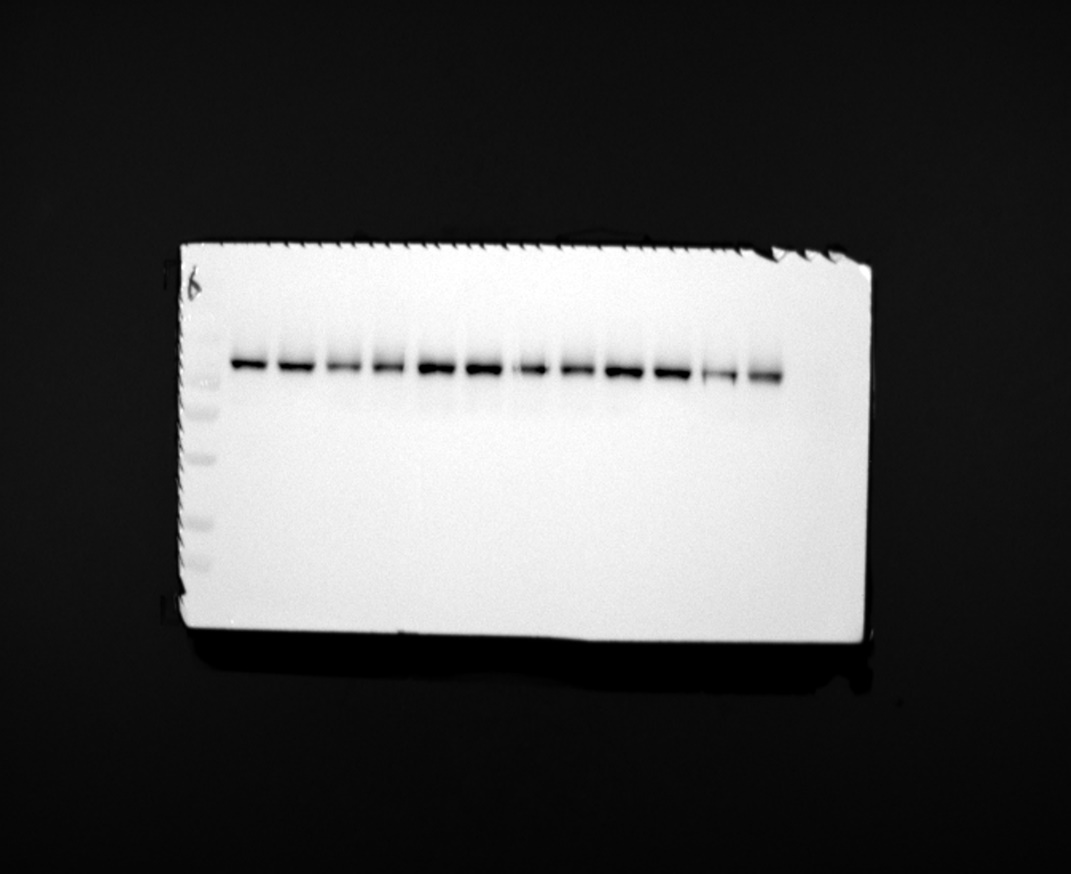


Fig s2-SK-N-BE(2)-IGF2BP3


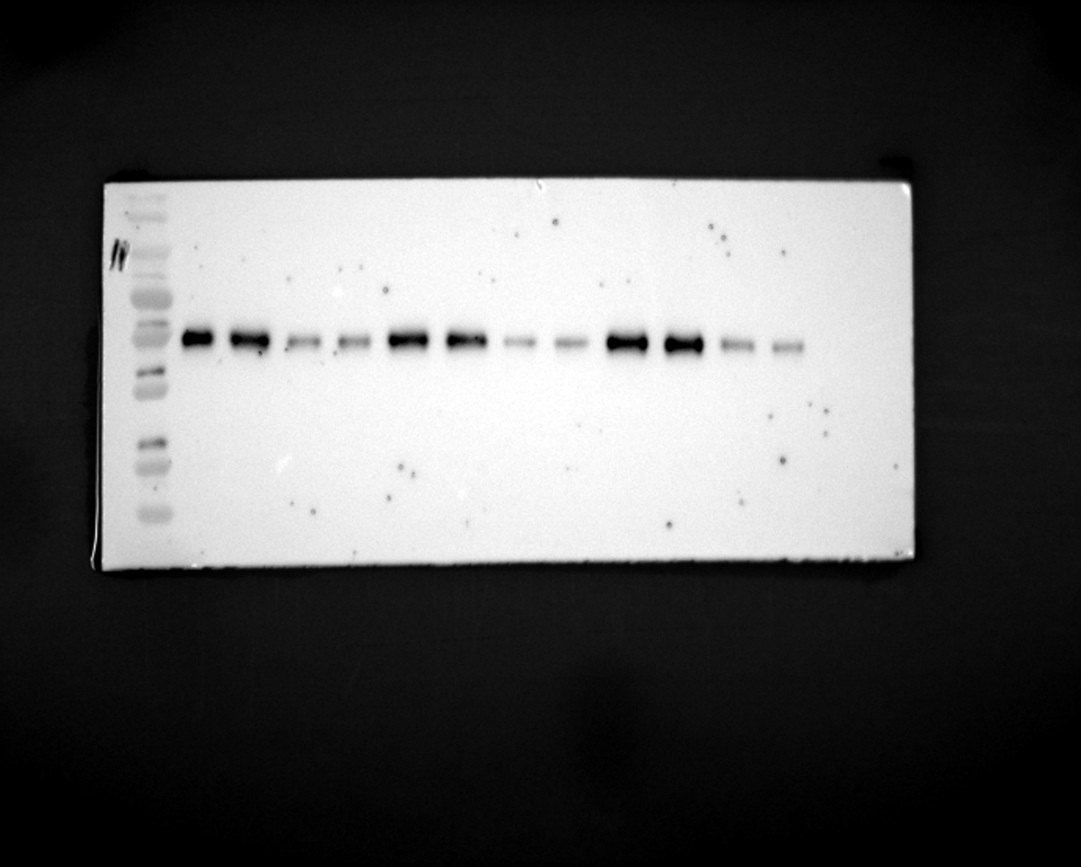


Fig s2-SK-N-BE(2)-N-Myc


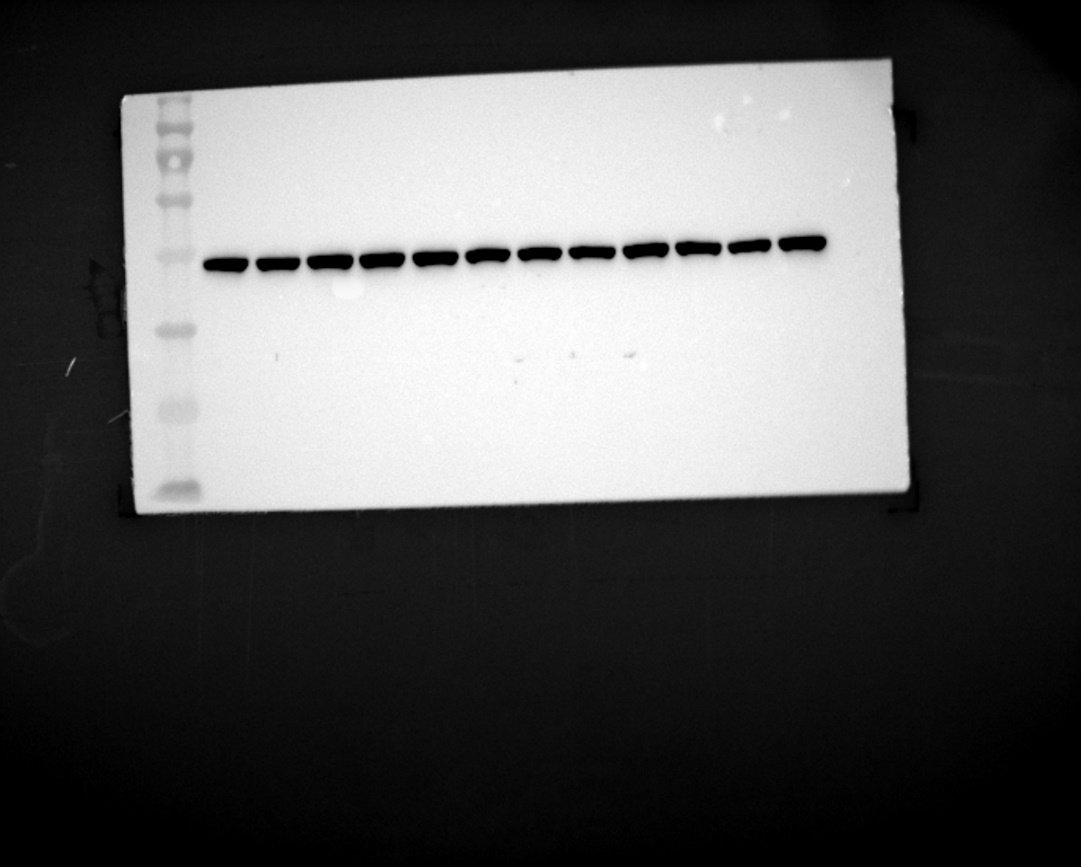


Fig s2-SK-N-BE(2)-β-actin


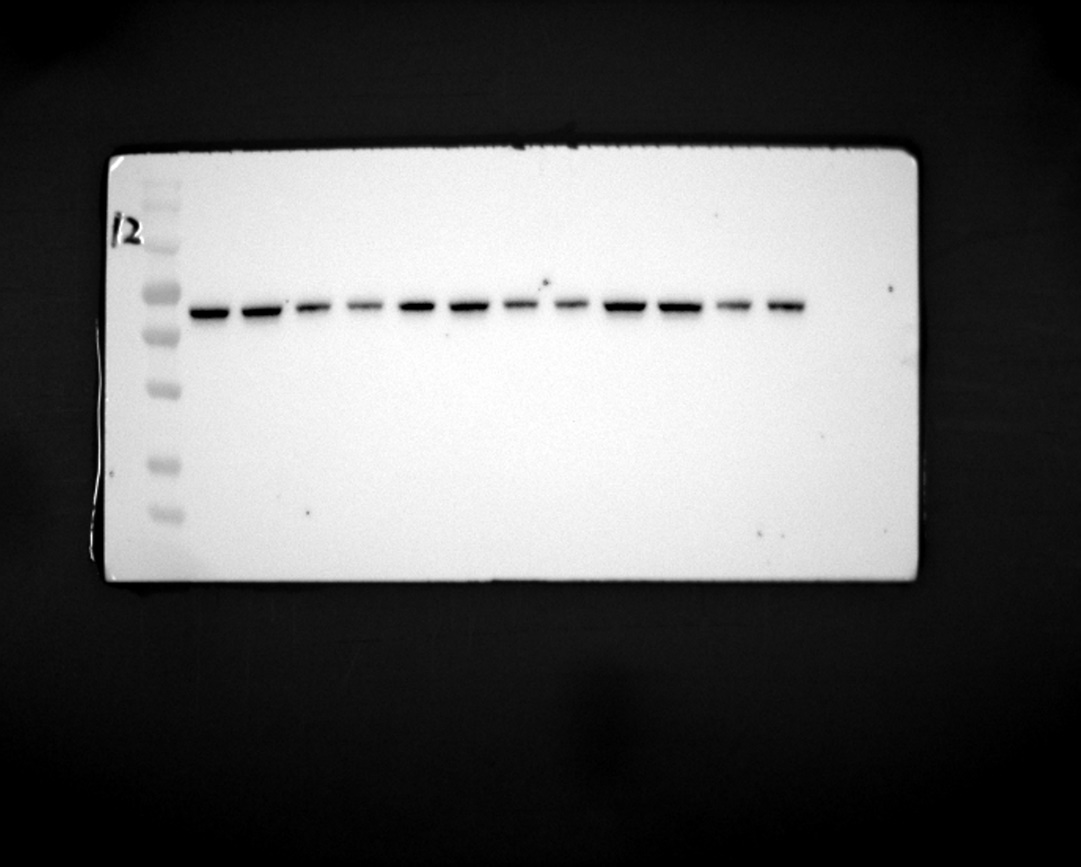


Fig s2-BE(2)-C-METTL3


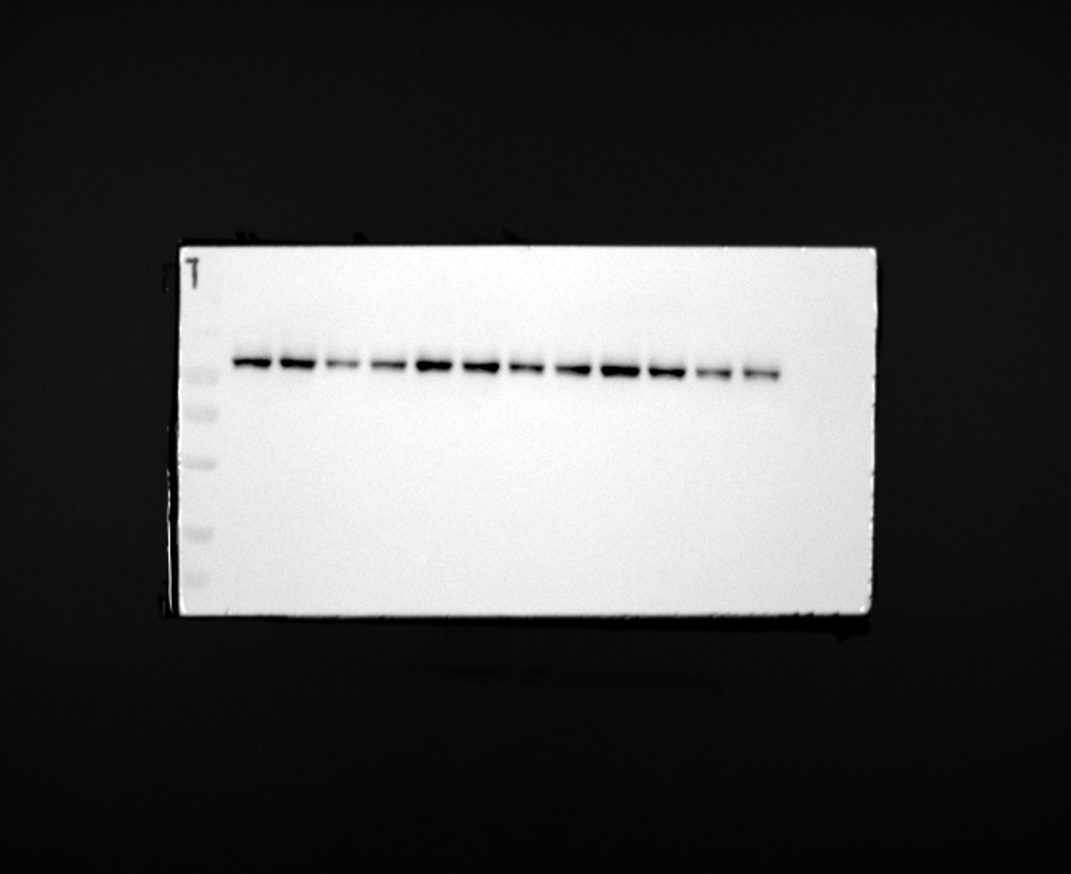


Fig s2-BE(2)-C-IGF2BP3


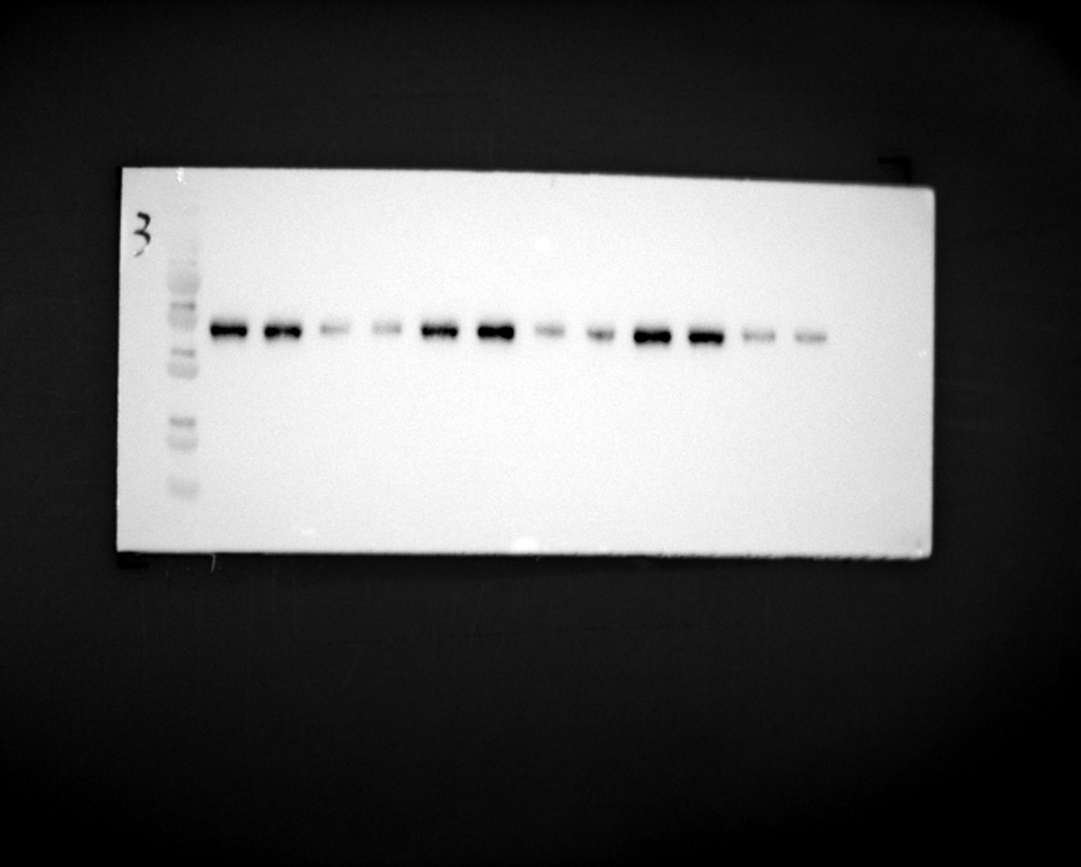


Fig s2-BE(2)-C-N-Myc


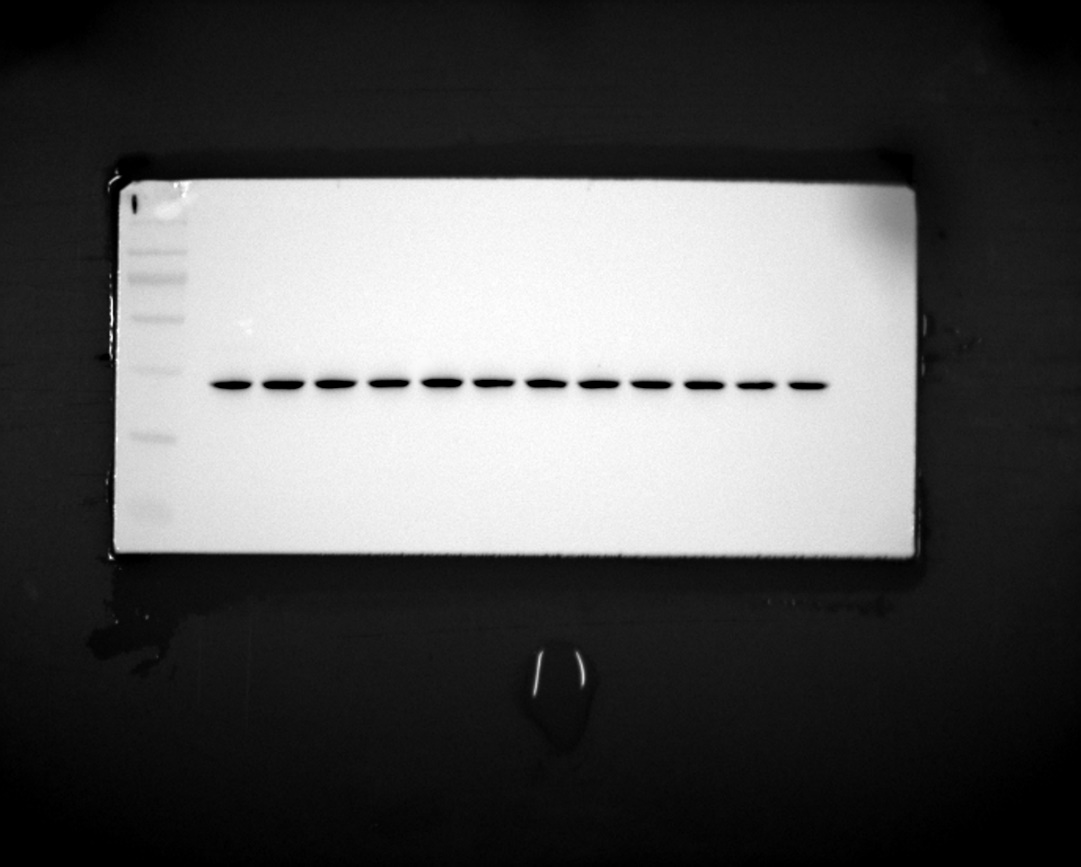


Fig s2-BE(2)-C-β-actin


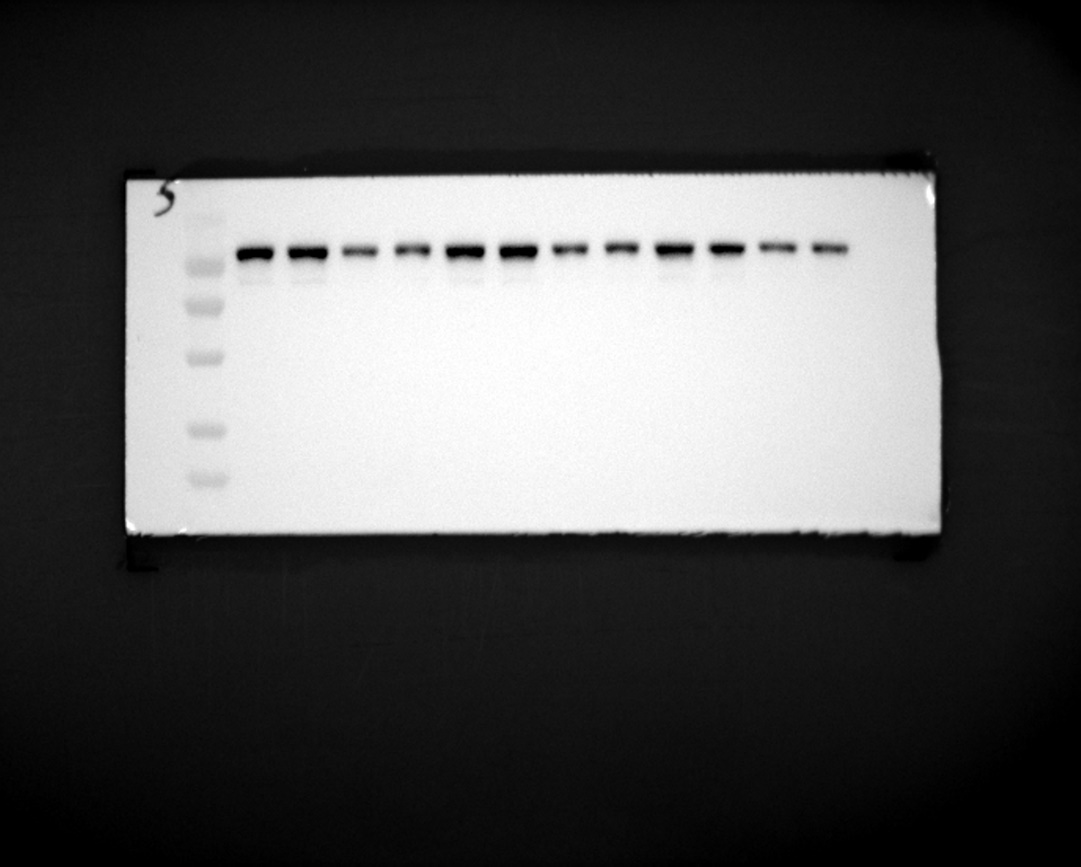


Fig s4-SK-N-BE(2)-IGF2BP3


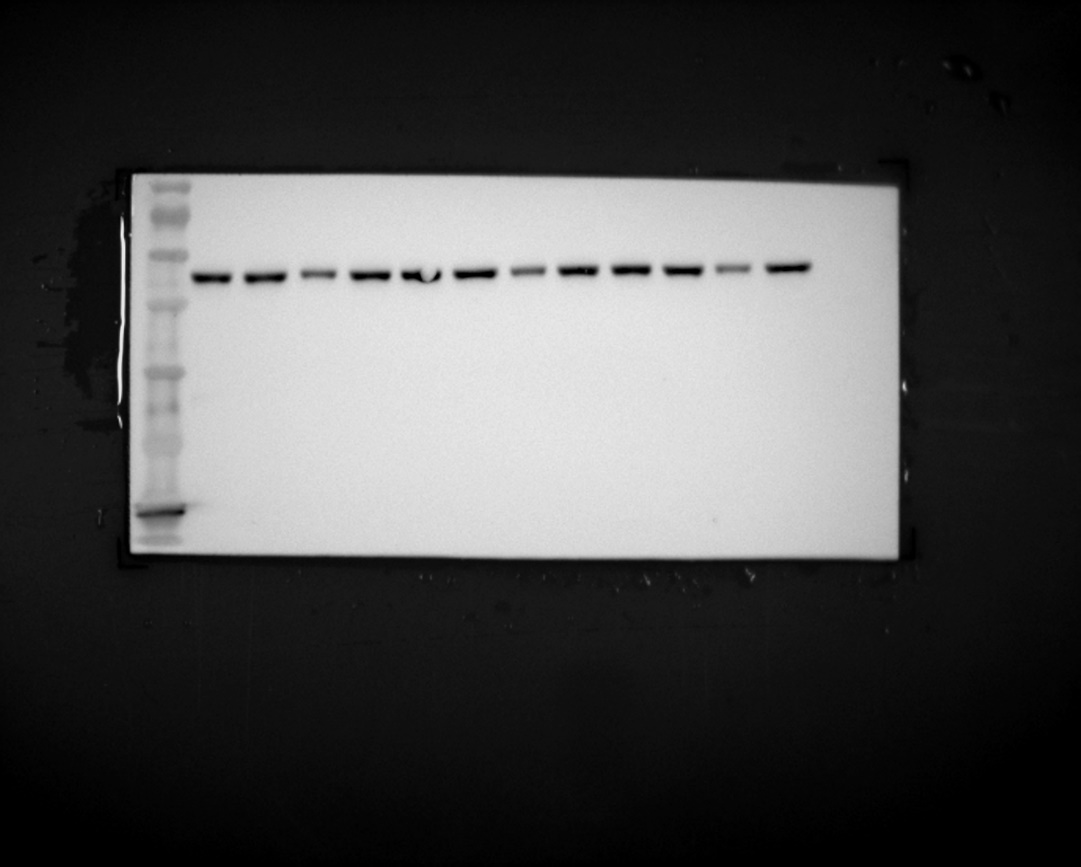


Fig s4-SK-N-BE(2)-N-Myc


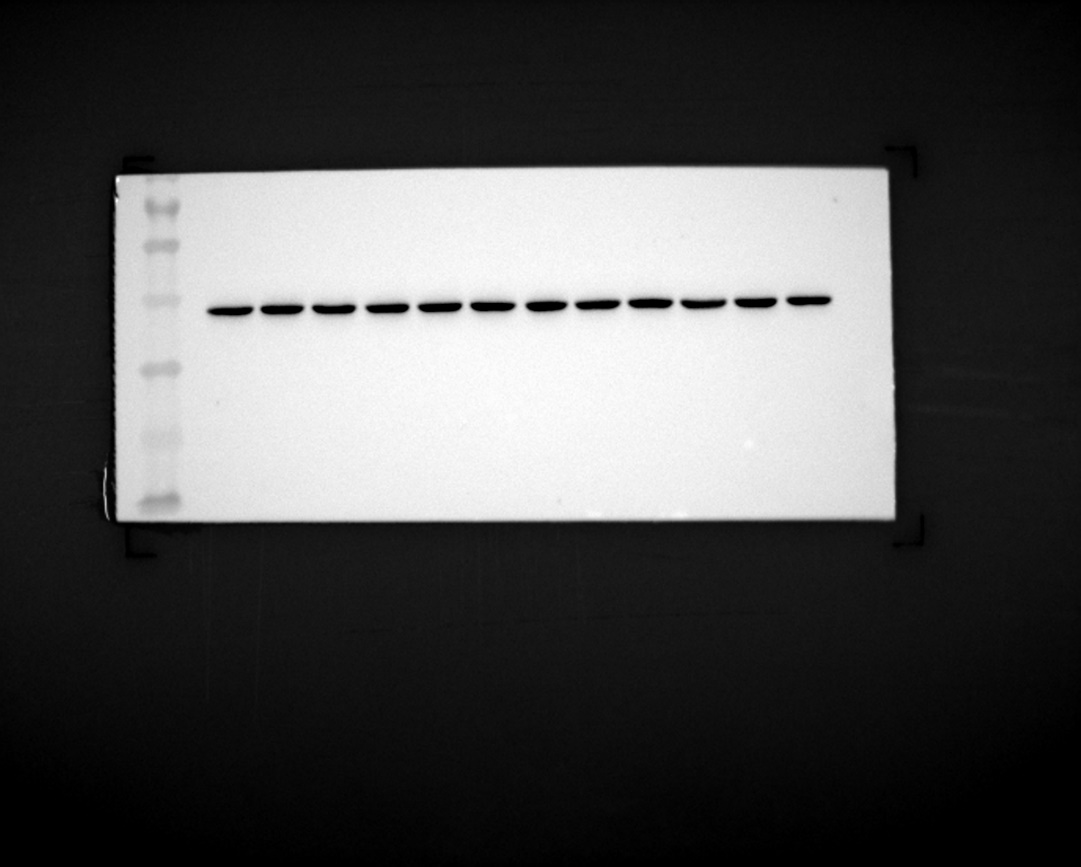


Fig s4-SK-N-BE(2)-β-actin


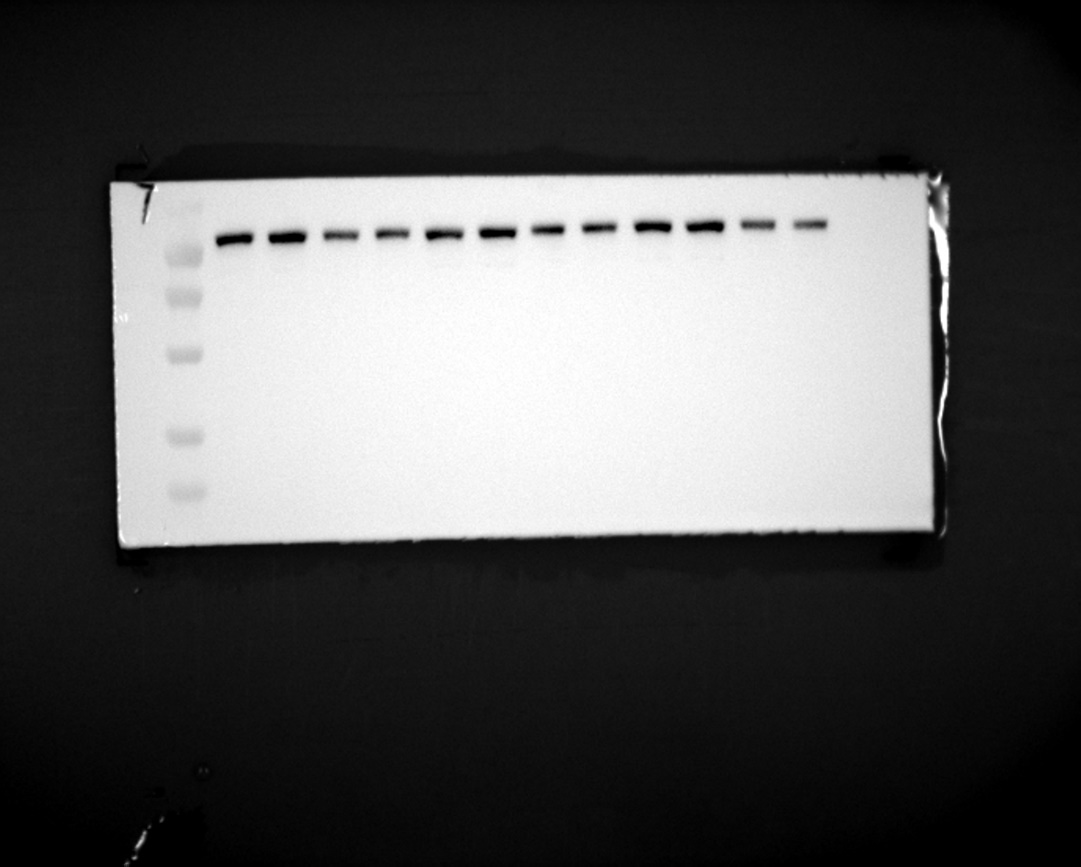


Fig s4-BE(2)-C-IGF2BP3


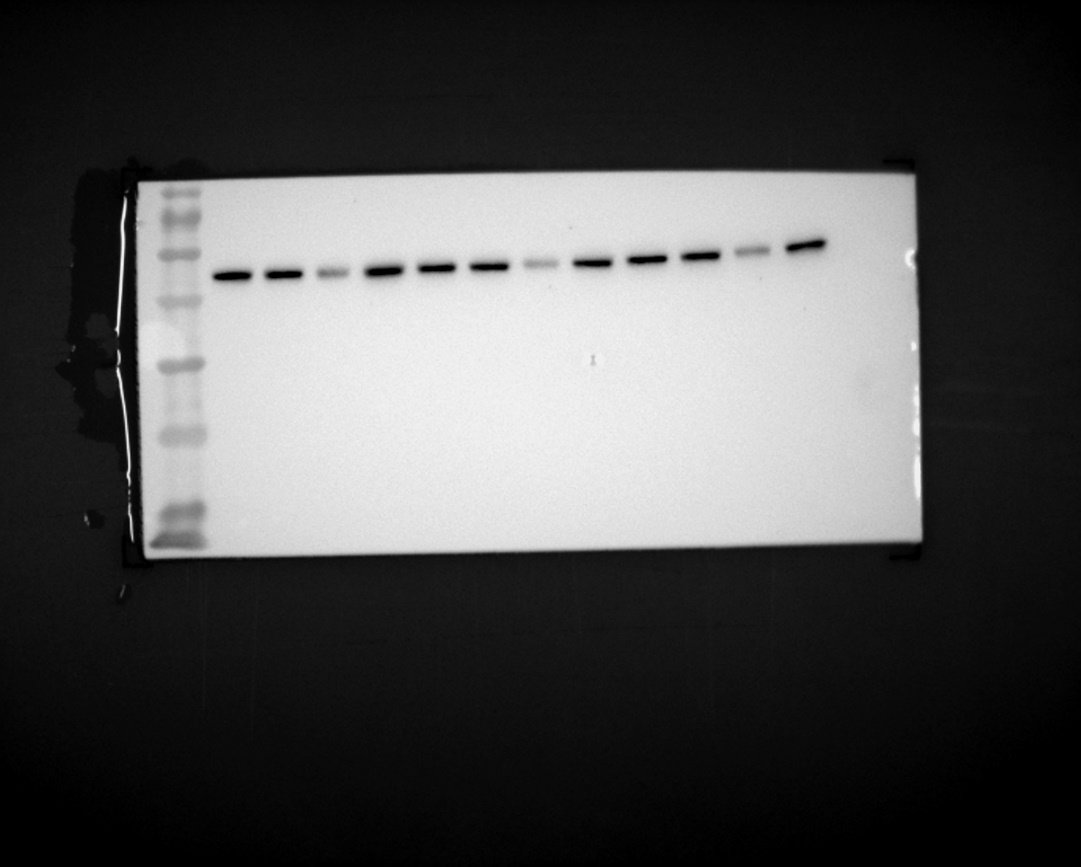


Fig s4-BE(2)-C-N-Myc


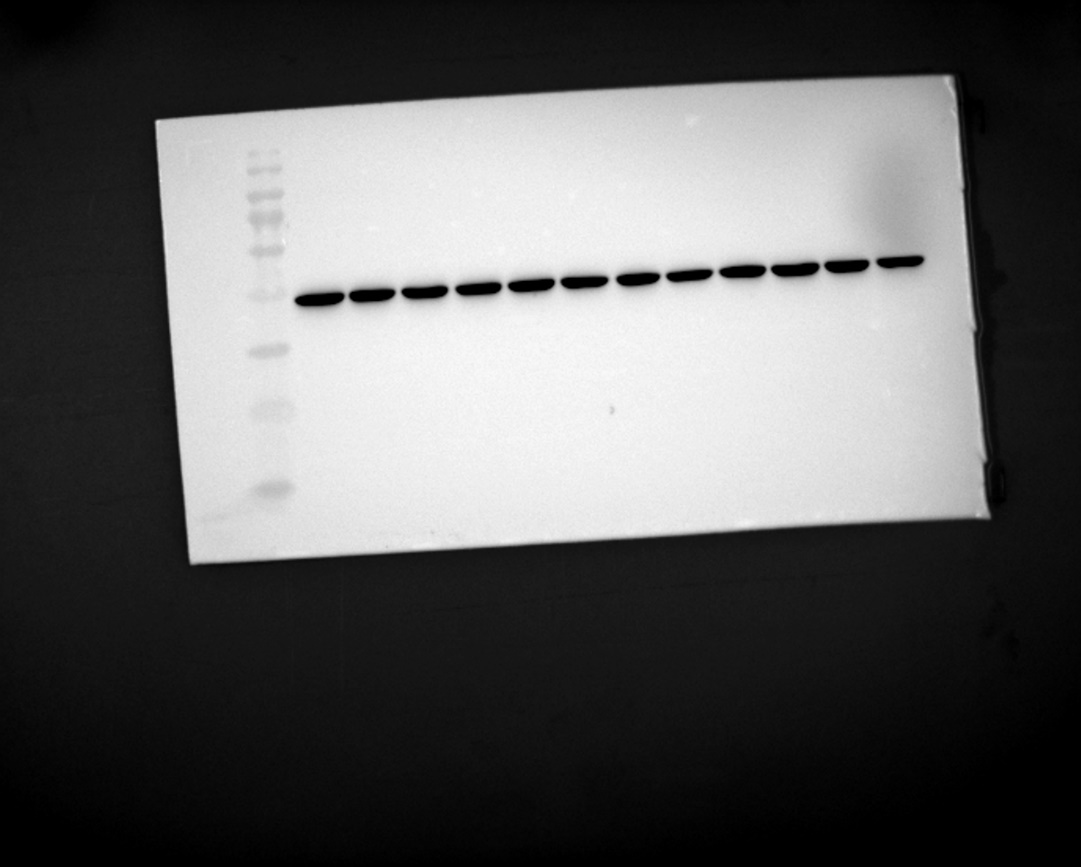


Fig s4-BE(2)-C-β-actin
